# Wavelet-Based P-Wave Detection in High-Rate GNSS Data: A Novel Approach for Rapid Earthquake Monitoring in Tsunamigenic Settings

**DOI:** 10.3390/s25133860

**Published:** 2025-06-21

**Authors:** Ajat Sudrajat, Irwan Meilano, Hasanuddin Z. Abidin, Susilo Susilo, Thomas Hardy, Brilian Tatag Samapta, Muhammad Al Kautsar, Retno Agung P. Kambali

**Affiliations:** 1 Department of Geodesy and Geomatics, Faculty of Earth Science and Technology, Institut Teknologi Bandung, Jalan Ganesa 10, Bandung 40132, Indonesiathomas.hardy@bmkg.go.id (T.H.); 2 Indonesian Meteorology, Climatology and Geophysics Agency (BMKG), Jakarta 12710, Indonesia; 3 National Research and Innovation Agency (BRIN), Bogor 16911, Indonesia; 4 Indonesian Geospatial Information Agency (BIG), Cibinong 16911, Indonesia

**Keywords:** P-wave detection, high-rate GNSS, wavelet transform, dynamic thresholding, tsunamigenic earthquakes, seismic monitoring, early warning systems

## Abstract

Rapid and accurate detection of primary waves (P-waves) using high-rate Global Navigation Satellite System (GNSS) data is essential for earthquake monitoring and tsunami early warning systems, where traditional seismic methods are less effective in noisy environments. We applied a wavelet-based method using a Mexican hat wavelet and dynamic threshold to thoroughly analyze the three-component displacement waveforms of the 2009 Padang, 2012 Simeulue, and 2018 Palu Indonesian earthquakes. Data from the Sumatran GPS Array and Indonesian Continuously Operating Reference Stations were analyzed to determine accurate displacements and P-waves. Validation with Indonesian geophysical agency seismic records indicated reliable detection of the horizontal component, with a time delay of less than 90 s, whereas the vertical component detection was inconsistent, owing to noise. Spectrogram analysis revealed P-wave energy in the pseudo-frequency range of 0.02–0.5 Hz and confirmed the method’s sensitivity to low-frequency signals. This approach illustrates the utility of GNSS data as a complement to seismic networks for the rapid characterization of earthquakes in complex tectonic regions. Improving the vertical component noise suppression might further help secure their utility in real-time early warning systems.

## 1. Introduction

Rapid and accurate identification of seismic waves, particularly primary waves (P-waves), which are compressional seismic waves that travel fastest through the Earth and arrive first at monitoring stations, is a critical aspect of earthquake monitoring and tsunami early warning systems [1,2]. P-waves are the fastest-traveling seismic waves and are the first alarming signals for imminent rupture that can deliver precious seconds to minutes of action in the case of tsunamigenic events [3,4,5,6]. Traditional seismometers that have been developed over several decades for this purpose, along with analysis techniques such as the short-term average/long-term average (STA/LTA) method, have been designed to identify the arrival of waves [7,8,9]. However, these techniques tend to perform poorly in noisy environments or with non-traditional data, which reduces their suitability for real-time usage in geologically complex tectonic settings.

Recent advances in global navigation satellite system (GNSS) data at high sampling rates have become an attractive addition to traditional seismic networks. In contrast to seismometers that monitor ground acceleration or velocity, high-attention GNSS stations record accurate surface displacements at a sampling rate of 1 Hz or higher, providing a direct view of the coseismic ground movements generated by large earthquakes [10,11,12,13,14]. This may be particularly useful for tsunamigenic earthquakes, which could benefit greatly from the rapid characterization of fault rupture and surface motion for use in tsunami generation models. The capability of a GNSS to sense seismic waves, including the arrival of P-waves, in near real-time, has been illustrated in studies to improve the areal coverage and reliability of early warning systems [14,15,16]. Nonetheless, the exploitation of GNSS data for automatic wave arrival detection remains limited, which is primarily related to the difficulties in recognizing weak P-wave phases with respect to noise and instrumental artifacts.

Seismic detection methods developed for broadband seismometers are not always suitable for GNSS time series. For example, the short-term average/long-term average (STA/LTA) algorithm, which relies on amplitude changes, struggles with low signal-to-noise ratio (SNR) GNSS data, leading to poor or incorrect detection [9]. Alternative methods, such as those based on the Akaike Information Criterion (AIC), evaluate statistical changes in waveforms, whereas the Hilbert–Huang transform (HHT) decomposes non-stationary signals [17,18]. Fast discrete wavelet transforms have also been used for seismic phase picking, offering computational efficiency, but requiring precise parameter tuning [19]. Akram and Eaton (2016) [19] provide a comprehensive review of arrival-time picking methods and highlight their challenges in low-SNR environments. Wavelet-based approaches, utilizing the Continuous Wavelet Transform (CWT), offer a robust method for decomposing signals into the time-frequency domain, enabling the detection of transient features such as P-wave onsets [17,18]. For instance, Zhang et al. (2003) [17] applied multiscale wavelet analysis to single-component seismic recordings, achieving reliable P-wave detection in high-SNR seismic data, whereas Shang et al. (2018) [18] combined wavelet denoising with kurtosis picking for seismometer waveforms. However, these methods are optimized for seismometer data and are less effective for high-rate GNSS displacement data, which exhibit a lower SNR and require dynamic thresholding to handle diverse noise conditions across different earthquake types and tectonic settings [20,21,22].

The present study fills these gaps by developing a new approach for detecting P-wave arrivals in the high-rate GNSS recordings of tsunamigenic earthquakes. We used CWT with the Mexican hat wavelet and dynamic thresholding method, which adjusts to local noise variations and is reliable for the precise identification of the P-wave onset [20,21,22]. When applied to actual GNSS time series for large tsunamigenic events, our method achieved better sensitivity and noise tolerance than existing methods. Through the use of a powerful combination of a GNSS and such processing, this research will also further integrate geodetic measurements into seismic monitoring systems, with an immediate impact on tsunami early warning and characterization of the earthquake source.

## 2. Materials and Methods

### 2.1. Data Acquisition and Sources

**Figure 1:**
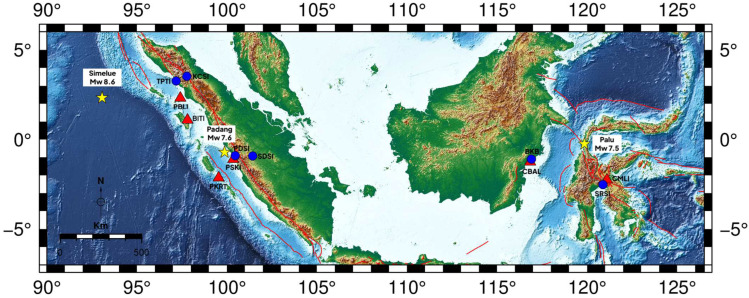
Map showing GNSS stations (red triangles), BMKG seismic stations (blue circles), earthquake epicenters (yellow stars), and major active fault lines (red lines) for the 2009 Padang, 2012 Simeulue, and 2018 Palu earthquakes. Distances are in kilometers, and a compass indicates orientation.

This study analyzed high-rate global navigation satellite system (GNSS) data from three tsunamigenic earthquakes in Indonesia: the 2009 Padang (Mw 7.6), 2012 Simeulue (Mw 8.6), and 2018 Palu (Mw 7.5) earthquakes, selected for their diverse tectonic settings, including megathrust subduction (Padang and Simeulue) and strike-slip faulting (Palu). Figure 1 illustrates the study area, showing the GNSS stations (red triangles) and the Indonesian Agency of Meteorological, Climatological, and Geophysical (BMKG) seismic stations (blue circles), with earthquake epicenters marked by yellow stars and major active fault lines indicated by red lines based on tectonic and geological mapping. The GNSS data for Padang and Simeulue were sourced from the Sumatran GPS Array (SuGAr), managed by the Earth Observatory of Singapore, and for Palu from the Indonesian Continuously Operating Reference Stations (InaCORS), operated by the Indonesian Geospatial Information Agency (Badan Informasi Geospatial, BIG). Ground displacements recorded at 1 Hz in the Receiver Independent Exchange (RINEX) format enabled high-resolution coseismic deformation analysis. The GNSS and seismic data supported wavelet-based P-wave arrival time detection and performance evaluation across tectonic settings.

### 2.2. GNSS Data Processing

High-rate GNSS data were processed using Precise Point Positioning with Ambiguity Resolution (PPP-AR) via PRIDE PPP-AR software (version 3.0, https://github.com/PrideLab/PRIDE-PPPAR, accessed on 3 November 2024) [23], chosen for its high-precision displacement estimates without nearby reference stations, which is ideal for Indonesia’s remote regions. Raw RINEX files were corrected with the International GNSS Service (IGS) final products (e.g., SP3 orbits and clock products) from Wuhan University (ftp://igs.gnsswhu.cn/pub/whu/phasebias/) and multi-GNSS observations (GPS, GLONASS) at 1 Hz. The workflow included least-squares position estimation, iterative outlier removal, and ambiguity resolution to achieve a real-time accuracy comparable to that of the IGS reference frame. The resulting three-component (East, North, Up) displacement time series were converted to the MiniSEED format using ObsPy (version 1.4.0) [24,25] for compatibility with seismic waveform analysis tools.

### 2.3. P-Wave Detection Using Dynamic Wavelet Thresholding

A wavelet-based method using a continuous wavelet transform (CWT) with a Mexican hat wavelet was developed to detect P-wave arrivals in three-component (East, North, Up) GNSS displacement waveforms, and validated against BMKG seismic records from stations proximate to or at comparable distances from GNSS stations. Implemented in Python with NumPy (version 3.9.4) [26], Matplotlib (version 1.26.4) [27], PyWavelets (version 1.3.0) [28], and ObsPy (version 1.4.0) [24,25], the method decomposes signals into time-frequency representations, defined as:

(1) $$W\left(a,b\right)=\frac{1}{\sqrt{a}}\int_{-\infty }^{\infty }x\left(t\right)\psi^{*}\left(\frac{t-b}{a}\right)dt\;$$

where $\left(W\left(a,b\right)\right)$ as the wavelet coefficient, (*a*) is the scale parameter, (*b*) as the time shift, is the input GNSS displacement signal, and $\psi^{*}\left(t\right)$ as the complex conjugate of the Mexican hat wavelet, defined as:

(2) $$\psi \left(t\right)=\frac{2}{\sqrt{3}\pi^{1/4}}\left(1-t^{2}\right)e^{-t^{2}/2}$$

This wavelet is applied within the continuous wavelet transform (CWT), as shown in Equation (1) [29]. The Mexican hat wavelet was chosen for its symmetric shape and compact time-domain support, which provides high sensitivity to transient signal changes, such as abrupt P-wave onsets in high-rate GNSS data [20,22]. Compared with the Morlet wavelet, which achieves optimal time-frequency resolution by approaching the Heisenberg uncertainty limit [29,30], the narrower time-domain support of the Mexican hat wavelet enhances its ability to localize short-duration transients in noisy GNSS data affected by atmospheric and multipath noise [31,32]. The scale parameter (a) is defined on a linear grid ranging from 1 to 50, corresponding to pseudo-frequencies from approximately 30 Hz to 0.6 Hz at a 1 Hz sampling rate, calculated using $f=\frac{f_{c}}{a\cdot \Delta t}\;$, where $f_{c}\approx 0.25$ is the center frequency of the Mexican hat wavelet, and Δt=1 s is the sampling interval [29,30]. This scale range ensures sufficient frequency resolution to capture P-wave signals in the expected frequency band. The decision rule for P-wave detection identifies the earliest time when the wavelet power in the East or North components exceeds the dynamic threshold. The dynamic threshold was calculated as follows:

(3) $$T_{dyn}\left(t\right)=\mu_{pre}+k\cdot \sigma_{roll}\left(t\right)$$

where $\mu_{pre}$ is the pre-event mean power, k=3.0 is the sensitivity factor, and $\sigma_{roll}\left(t\right)$ is the rolling standard deviation of the wavelet power that distinguishes P-wave signals from noise [30,31]. To optimize the sensitivity factor *k* = 3.0, we tested values from 2.0 to 4.0 and found that *k* = 3.0 balanced false positives and detection sensitivity across all events. Lower values (e.g., *k* = 2.0) increased false detections in noisy data, whereas higher values (e.g., *k* = 4.0) miss weak P-wave signals. Future work will investigate adaptive or data-driven thresholding models to dynamically adjust (*k*) based on the station-specific noise characteristics [33,34]. Given the robustness of the East and North components in detection, the Up component is considered only when noise levels are low, owing to its susceptibility to ionospheric, tropospheric, and multipath noise [31]. The implementation utilized Python libraries (NumPy [26], PyWavelets [28], and ObsPy [24,25]) with custom configurations to ensure robust detection tailored to the GNSS data characteristics. P-wave arrival times were identified when the wavelet power in the East or North components exceeded the threshold before the maximum wavelet power peak, leveraging their sensitivity to compressional waves. Validation against the BMKG InaTEWS earthquake repository calculated the time differences as follows:

(4) $$\Delta t=t_{arrival\;GNSS\;}-t_{arrival\;seismic}$$

The results are visualized in three-panel plots (waveform, wavelet power, spectrogram) per station and component to assess the performance across events.

## 3. Results

**Figure 2:**
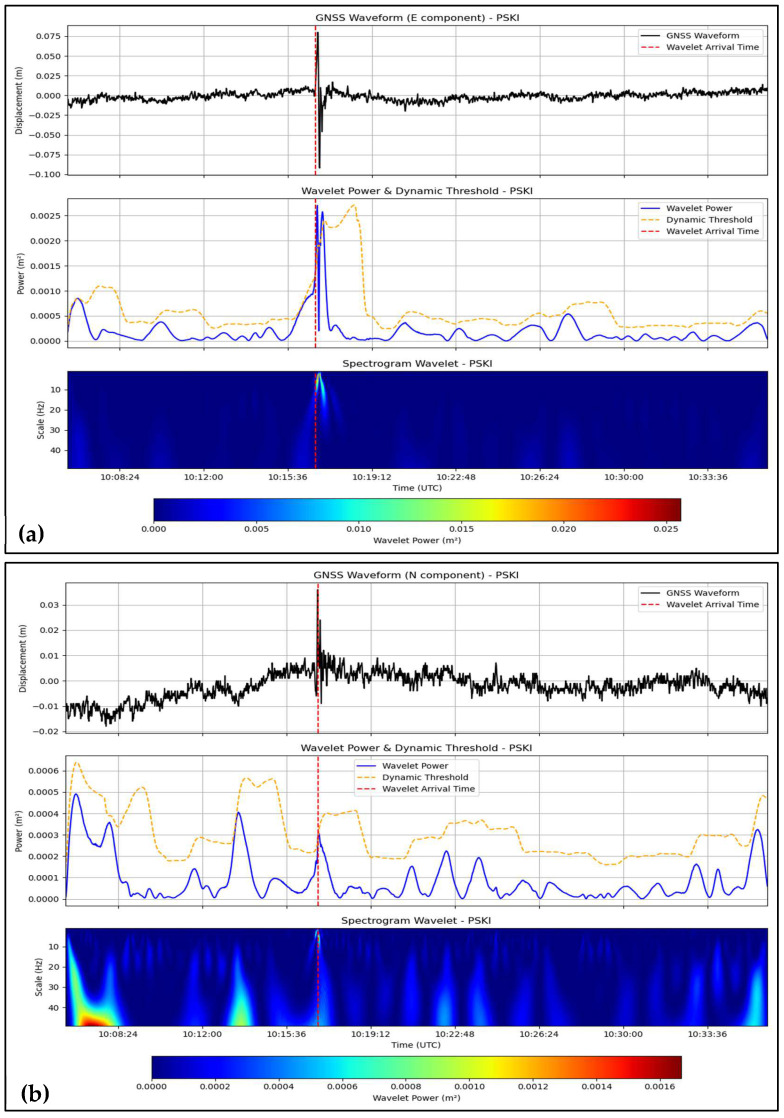
Wavelet-based P-wave detection for the 2009 Padang earthquake (Mw 7.6) at PSKI station. (**a**) East (E) component waveform at PSKI (69.62 km from epicenter), with P-wave arrival at 10:16:47 UTC (red dashed line), wavelet power (blue line), and dynamic threshold (yellow dashed line). (**b**) North (N) component at PSKI, arrival at 10:16:56 UTC. Wavelet power peaks indicate robust P-wave signals, validated against BMKG seismic records (Table 1). Data for PKRT station are presented in Supplementary Figure S1.

**Figure 3:**
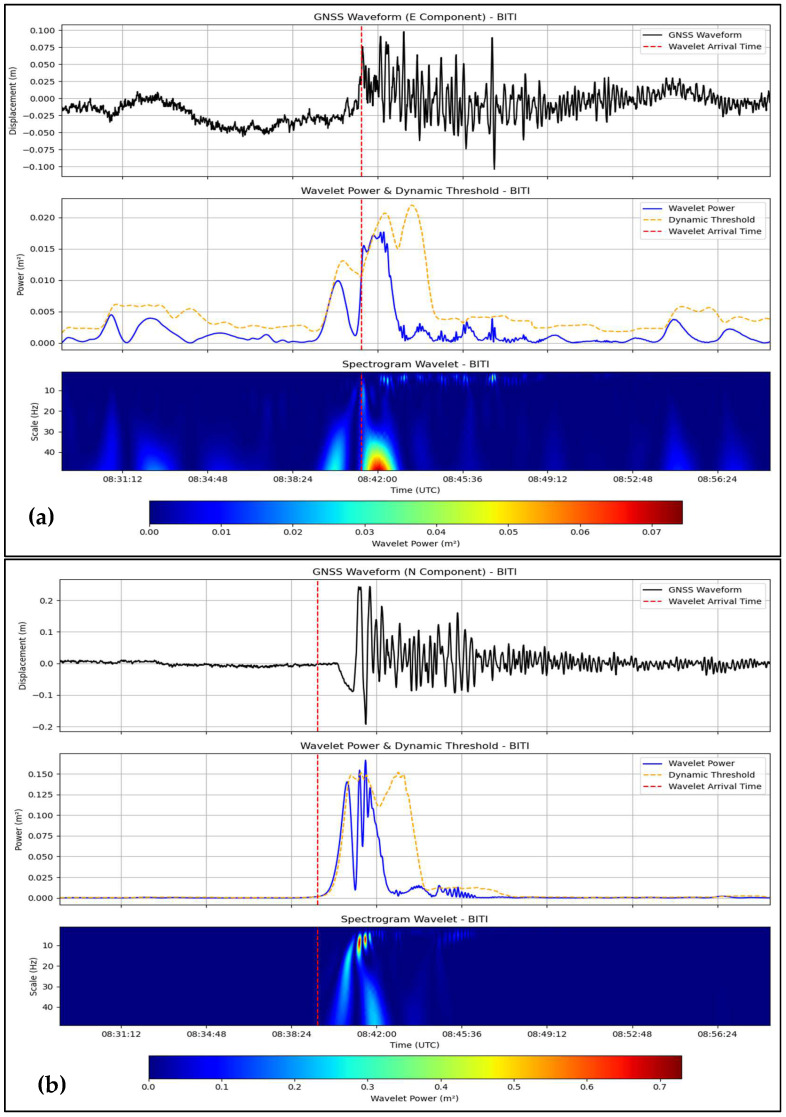
Wavelet-based P-wave detection for the 2012 Simelue earthquake (Mw 8.6) at BITI station. (**a**) East (E) component waveform at BITI (545.94 km from epicenter), with P-wave arrival at 08:41:19 UTC (red dashed line), wavelet power (blue line), and dynamic threshold (yellow dashed line). (**b**) North (N) component at BITI, arrival at 08:39:30 UTC. Wavelet power peaks indicate robust P-wave signals, validated against BMKG seismic records (Table 1). Data for PBLI station are presented in Supplementary Figure S2.

**Figure 4:**
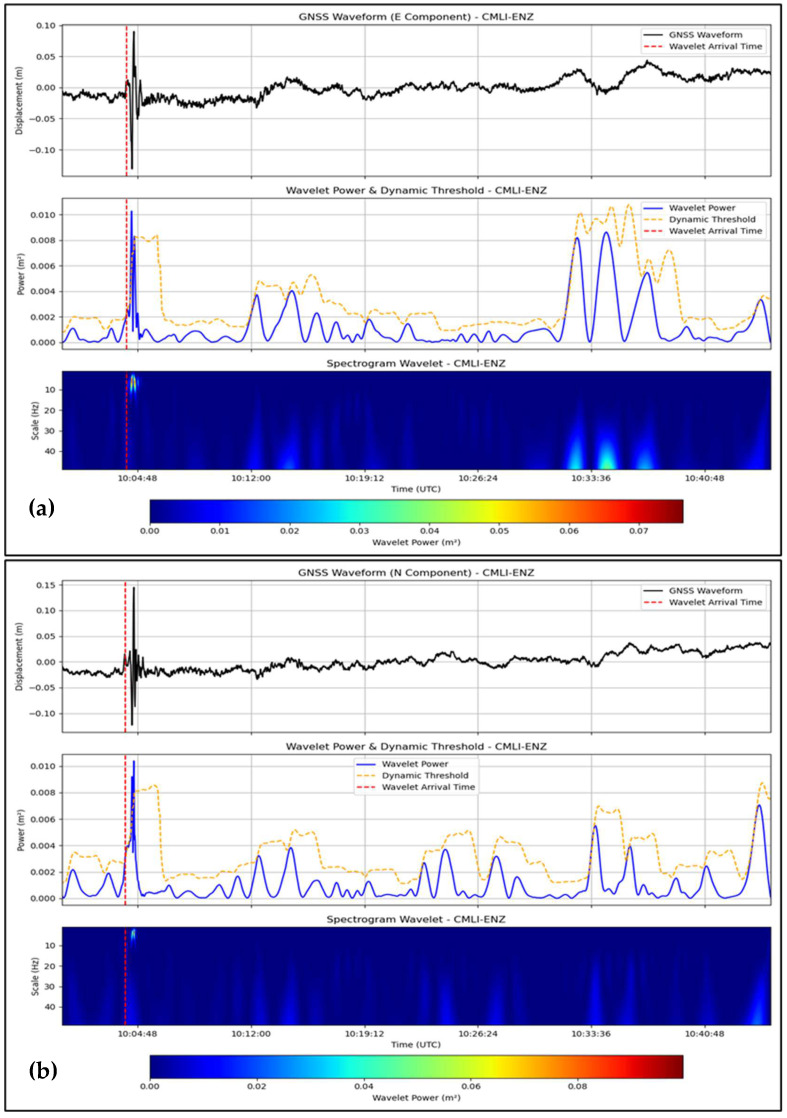
Wavelet-based P-wave detection for the 2018 Palu earthquake (Mw 7.5) at CMLI station. (**a**) East (E) component waveform at CMLI (297.62 km from epicenter), with P-wave arrival at 10:04:07 UTC (red dashed line), wavelet power (blue line), and dynamic threshold (yellow dashed line). (**b**) North (N) component at CMLI, arrival at 10:04:00 UTC. Wavelet power peaks indicate robust P-wave signals, validated against BMKG seismic records (Table 1). Data for CMLI station are presented in Supplementary Figure S3.

Based on the three-component (East, North, Up) high-rate ground displacement waveforms from the 2009 Padang (Mw 7.6), 2012 Simeulue (Mw 8.6), and 2018 Palu (Mw 7.5) earthquakes in Indonesia, this wavelet-based approach was combined with the continuous wavelet transform (CWT), using the Mexican hat wavelet and dynamic thresholding method. The P-wave arrival times were compared with the seismic arrival times recorded by the BMKG seismic records to cross-validate them by matching the records from the nearest seismometers: PDSI (Padang), TPTI (Simeulue), and SRSI (Palu) for the GNSS-based P-wave arrival times from PSKI, PBLI, and CMLI, respectively. The results are shown in Table 1 and Figure 2, Figure 3 and Figure 4, allowing us to conclude that the method is effective for the East and North components but not the Up component, despite differences in the tectonic settings of Padang and Simeulue (megathrust subduction) compared to Palu (strike-slip). The wavelet power results confirmed the P phase in the 0.02–0.5 Hz pseudo-frequency band as a prominent characteristic for near-field (e.g., PSKI, 69.62 km) and regional (e.g., PBLI, 483.33 km) stations.

### 3.1. Padang Earthquake—30 September 2009 (Mw 7.6)

The onset of the P-wave at PSKI (69.62 km) and PKRT (162.26 km) was determined from the GNSS data. At PSKI, the East (E) and North (N) components showed strong wavelet power peaks exceeding the dynamic threshold at 10:16:47 UTC and 10:16:56 UTC, respectively (Figure 2a,b). Verification with PDSI (10:16:26 UTC, 66.6 km) currently has time lags of +21.0 s (E) and +30.0 s (N) (Table 1). At PKRT, both the E and N waveforms of the arrivals at 10:16:10 UTC were clear, and their power peaks were obvious (Supplementary Figure S1). With respect to SDSI (177.6 km, 10:16:37.6 UTC), the discrepancies were −27.6 s (E) and −27.6 s (N). U at both stations yielded bad onsets (e.g., −585.0 s at PSKI, −593.6 s at PKRT, Table 1), where the low power of the wavelet suggested noise-contaminated data. The measurements of the E and N components were accurate within ±30 s, which demonstrates the reliability of the detection of near-field and intermediate-distance locations.

### 3.2. Simeulue Earthquake—11 April 2012 (Mw 8.6)

The P-wave arrival was also determined using PBLI (483.33 km) and BITI (545.94 km) data. The 08:38:37 UTC and 08:39:12 UTC PBLI arrivals of E and N (respectively) displayed well-separated wavelet power peaks for P (Figure 3a,b). The comparison against TPTI (466.2 km, 08:39:37.2 UTC) yielded deviations of −60.2 s and −25.2 s (Table 1). The UTC 08:41:19 (E) and 08:39:30 (N) arrivals observed at BITI had a high SNR and strong signals (Supplementary Figure S2). The offsets with respect to KCSI (543.9 km) were +91.1 s and −17.9 s. The U component was noisiest (+79.8 s at PBLI, +876.1 s at BITI). The E and N components were well suited for regional detection up to ±90 s.

### 3.3. Palu Earthquake—28 September 2018 (Mw 7.5)

We considered the GNSS observations of the CMLI (297.62 km) and CBAL (352.43 km). The E and N onsets at CMLI (Figure 4a,b) had relatively strong wavelet power peaks at 10:04:07 UTC and 10:04:00 UTC, respectively. For the validation using SRSI (277.5 km, 10:03:24.9 UTC), they were +42.1 s (E) and +35.1 s (N) (Table 1). The onset time of the N component on CBAL at 10:04:39 UTC was stable (Supplementary Figure S3), where the time differences with BKB (344.1 km, 10:03:31.2 UTC) were +67.8 s. The onset times of the E component at CBAL (+204.8 s) and U components at the two stations (−154.9 s on CMLI, +201.8 s on CBAL) were less stable and might have been affected by site effects and the signal losses. The accuracy of the N component (+35.1 s to +67.8 s) shows that the method also works for intermediate distances.

The wavelet-based algorithm detected P-wave arrivals in the East and North components for all events with time differences of ±90 s to the BMKG seismic records (Table 1) in the context of quick earthquake monitoring. The Up component revealed untrustworthy onsets across all stations (e.g., −585.62 s at PSKI, +79.8 s at PBLI, +201.8 s at CBAL, Table 1), indicating high noise amplification and low responsiveness to P-waves due to ionospheric, tropospheric, and multipath effects (Supplementary Figures S1–S3). Wavelet power results confirmed the P phase in the 0.02–0.5 Hz pseudo-frequency band as a prominent characteristic for near-field (e.g., PSKI, 69.62 km) and regional (e.g., PBLI, 483.33 km) stations. The larger discrepancies at intermediate distances (e.g., CBAL, +204.8 s, Table 1) suggest signal attenuation, particularly for the East component in the strike-slip regime of Palu.

To provide insight into the input data for our wavelet-based P-wave detection, Supplementary Figure S4 presents the raw GNSS displacement time series (East, North, Up components) for the 2009 Padang earthquake at PSKI (69.62 km from the epicenter), the 2012 Simeulue earthquake at PBLI (483.33 km from epicenter), and the 2018 Palu earthquake at CMLI (297.62 km from epicenter), alongside the corresponding seismometer velocity waveforms from the PDSI (Padang, 66.6 km), TPTI (Simeulue, 466.2 km), and SRSI (Palu, 277.5 km) stations. These visualizations illustrate the complementary nature of the GNSS absolute displacements and seismometer velocity records, supporting the validation of our P-wave detection results.

### 3.4. Comparison with STA/LTA Method

To evaluate the robustness of our wavelet-based method, we compared its performance with the classical STA/LTA method applied to the North component of GNSS data at PSKI (2009 Padang earthquake, Mw 7.6) and PBLI (2012 Simeulue earthquake, Mw 8.6). The STA/LTA algorithm was configured with a short-term window of 5 s, a long-term window of 10 s, and a threshold of 3.0 [31]. For PSKI (69.62 km from epicenter), our wavelet method detected the P-wave onset at 10:16:56 UTC (Table 1), consistent with BMKG seismic records (PDSI, 10:16:26 UTC, +30 s offset), whereas STA/LTA produced an inaccurate detection at 10:07:55 UTC (−522.66 s offset) due to multipath noise (Supplementary Figure S5).

For PBLI (483.33 km), the wavelet method accurately detected the P-wave at 08:39:12 UTC (Table 1), whereas STA/LTA failed to identify the onset, resulting in a false negative result (Supplementary Figure S6). These results demonstrate that our wavelet-based method, with its dynamic thresholding, outperforms STA/LTA in low-SNR GNSS data, where static thresholding struggles with noise variations [9,31].

## 4. Discussion

In this study, a continuous wavelet transform (CWT) with a Mexican hat wavelet was used to detect P-wave arrivals in a high-rate global navigation satellite system (GNSS) time series, testing the hypothesis that a GNSS can complement seismic networks in tsunamigenic regions for real-time earthquake monitoring. We applied this method to the 2009 Padang (Mw 7.6), 2012 Simeulue (Mw 8.6), and 2018 Palu (Mw 7.5) earthquakes to assess its effectiveness, limitations, and broader impact. The positive detections of P-wave onsets in the East (E) and North (N) components, with deviations within ±90 s compared to seismic records from the Indonesian Agency for Meteorological, Climatological, and Geophysical (BMKG) (Table 1; Figure 2, Figure 3 and Figure 4), except for CBAL’s East component (+204.80 s), confirm the utility of the method for rapid earthquake detection and source characterization [9,30,35,36,37]. However, the ±90 s offset is too large for operational tsunami early warning systems, which require sub-second accuracy [3,6]. Latency primarily stems from GNSS data processing, specifically precise point positioning with ambiguity resolution (PPP-AR) processing via PRIDE PPP-AR software, which takes approximately 10–15 s per epoch on a standard computing setup [23]. For real-time applications, integrating cloud-based processing or pre-computed International GNSS Service (IGS) products could reduce latency to sub-10-s levels, enhancing the viability of the method for rapid monitoring and tsunami early warning [32,38].

Unlike the classical short-term average/long-term average (STA/LTA) method, which is susceptible to site-specific amplification and noise [9,31], the wavelet-based approach leverages absolute displacement waveforms to detect P-wave signals in the 0.02–0.5 Hz pseudo-frequency range across diverse tectonic settings, including megathrust subduction (Padang, Simeulue) and strike-slip faulting (Palu). A quantitative comparison with STA/LTA, detailed in Section 3.4, demonstrates the superiority of the wavelet method in low signal-to-noise ratio (SNR) environments. For instance, at PSKI (2009 Padang, 69.62 km), the wavelet method detected the P-wave onset at 10:16:56 UTC (North, +30 s offset), whereas STA/LTA yielded an inaccurate detection at 10:07:55 UTC (−522.66 s offset) due to multipath noise (Supplementary Figure S5). Similarly, at PBLI (2012 Simeulue, 483.33 km), the wavelet method accurately detected the P-wave at 08:39:12 UTC (North), whereas STA/LTA failed, resulting in a false negative (Supplementary Figure S6) [31,39,40]. This time-independent feature, described in Section 2.3, supports blind detection, which is a critical requirement for tsunami early warning systems.

The reliability of the E and N components was consistent in both near-field (e.g., PSKI, 69.62 km, ±30 s; Figure 2) and regional stations (e.g., BITI, 545.94 km, ±90 s; Figure 3), aligning with prior studies on GNSS coseismic displacement detection [9,30,35,36,37]. The wavelet’s performance in the 0.02–0.5 Hz range (Figure 2, Figure 3 and Figure 4) mirrors seismological applications for isolating transient signals [22,31]. However, significant deviations at greater distances (e.g., CBAL, +204.80 s East, +67.80 s North; Supplementary Figure S3) suggest signal attenuation, particularly in strike-slip settings like Palu, where wave diffusion may degrade GNSS signal quality.

The Up (U) component exhibited systematic and large time lags (e.g., −585.0 s at PSKI, +876.1 s at BITI, +201.8 s at CBAL; Table 1; Supplementary Figures S1–S3), confirming its inconsistent reliability. This issue, likely due to ionospheric, tropospheric, and multipath noise, as well as low vertical antenna sensitivity [31,39,40], persisted despite preprocessing steps, such as iterative outlier removal and least-squares position estimation during PPP-AR processing [23]. The low wavelet power and noise contamination indicate that the Up component is less sensitive to P-wave compressional signals than anticipated [31,39]. Dynamic thresholding (*k* = 3.0, Section 2.3) also failed to mitigate these effects, suggesting the need for component-specific noise mitigation strategies. To optimize the sensitivity factor *k* = 3.0, we tested values from 2.0 to 4.0 and found that *k* = 3.0 balanced false positives and detection sensitivity across all events. Lower values (e.g., *k* = 2.0) increase false detections in noisy data, whereas higher values (e.g., *k* = 4.0) miss weak P-wave signals. Future work should explore adaptive or data-driven thresholding models to dynamically adjust k based on the station-specific noise characteristics [33,34].

The hypothesis that a wavelet-based thresholding technique would outperform conventional seismic methods was partially validated using GNSS data. The robustness of the E and N components across various tectonic settings and distances highlights the superiority of the method over STA/LTA noise sensitivity [9,31]. However, the ±90 s accuracy, which is suitable for rapid monitoring, falls short of the sub-second precision achieved by seismic networks [3,6,41,42]. The unreliability of the Up component further limits the comprehensive detection. These findings suggest that a GNSS is most effective as a complement to seismic networks, particularly for large-magnitude events that may saturate seismometers, such as the Mw 8.6 Simeulue earthquake [15].

GNSS-based seismic monitoring is particularly valuable for tsunami-prone regions. The ability of this method to detect P-waves in the E and N components using unsaturated displacement data (Figure 2, Figure 3 and Figure 4) enables rapid source characterization, which is essential for tsunami early warning systems. Its applicability in subduction (Padang and Simeulue) and transform (Palu) environments suggests its potential for global use in regions such as Japan, Chile, and California. The 0.02–0.5 Hz P-wave energy band provides a reference for real-time algorithms, enhancing earthquake source models and early warning systems. By leveraging the globally available GNSS infrastructure, this method could improve monitoring in areas with sparse seismic networks and democratize access to disaster risk reduction tools.

Despite these strengths, this method has several limitations. The noise susceptibility of the Up component restricts its usability, necessitating advanced signal processing techniques, such as adaptive filtering, multi-station stacking, and machine learning-based signal classification [33,42,43,44,45,46,47]. The constant sensitivity factor (*k* = 3.0) may not adapt to site-specific noise variations (e.g., local geology or anthropogenic activity), potentially causing errors at greater distances (e.g., CBAL East, +204.80 s). Although adequate for rapid monitoring, the ±90 s accuracy is insufficient for sub-second real-time applications, and signal attenuation at larger distances complicates the consistent detection. To enhance generalizability, future research should test the method on GNSS data from crustal and extensional earthquakes in regions, such as Java, Indonesia, Japan, and Chile, across a broader magnitude range (e.g., Mw 5.0–6.5) and diverse faulting mechanisms [36,37]. Exploring alternative wavelet functions (e.g., Morlet) or variable sensitivity factors tailored to station-specific noise profiles can improve thresholding accuracy. Integrating multi-GNSS constellations (e.g., GPS, GLONASS, and Galileo) may enhance vertical precision and reduce errors [48,49,50]. Real-time implementation, combining cloud-based wavelet analysis with PPP-AR, can minimize latency, making the method viable for operational early warning systems.

## 5. Conclusions

This study introduces a wavelet-based method utilizing high-rate GNSS data for robust P-wave onset detection, achieving accurate detections in the East and North components across three significant Indonesian earthquakes: the 2009 Padang (Mw 7.6), 2012 Simeulue (Mw 8.6), and 2018 Palu (Mw 7.5). The method, employing the continuous wavelet transform (CWT) with a Mexican hat wavelet, yielded deviations within ±90 s compared to BMKG seismic records (Table 1), except for the CBAL East component (+204.80 s, Supplementary Figure S3), demonstrating its effectiveness for rapid earthquake detection and source characterization. A comparative analysis with the short-term average/long-term average (STA/LTA) algorithm highlights the wavelet method’s superiority in low signal-to-noise ratio (SNR) environments: at PSKI, the wavelet detected the P-wave at 10:16:56 UTC (+30.0 s offset) versus an inaccurate STA/LTA detection at 10:07:55 UTC (−522.66 s offset) due to multipath noise (Supplementary Figure S5), whereas at BITI, the wavelet identified the onset at 08:39:30 UTC (−17.9 s offset), where STA/LTA failed (Supplementary Figure S6).

However, the Up component remains unreliable, exhibiting significant time discrepancies (e.g., −585.0 s at PSKI, +876.1 s at BITI, +201.8 s at CBAL, Table 1), attributed to ionospheric, tropospheric, and multipath noise. The ±90 s latency, primarily owing to precise point positioning with ambiguity resolution (PPP-AR) processing, requiring approximately 10–15 s per epoch on a typical computing setup, limits its suitability for sub-second tsunami early warning systems. Future research should focus on advanced denoising techniques, such as adaptive filtering or multi-station stacking, to enhance Up-component reliability, integrate cloud-based processing or pre-computed IGS products to reduce latency to less than 10 s, and validate the method across diverse tectonic settings (e.g., crustal and extensional earthquakes in Java, Japan, and Chile) to broaden its generalizability for global earthquake monitoring and early warning applications.

## Figures and Tables

**Table 1:** Summary of P-wave arrival times and distances for the 2009 Padang, 2012 Simeulue, and 2018 Palu earthquakes at GNSS and BMKG seismic stations.

Events	Stations GNSS	Distance	Components	P Arrival Time (Wavelet)	Stations Seismic BMKG	Distance (Km)	P Arrival Time (Seismic)
Padang 30 September 2009 Mw 7.6	PSKI	69.62	E	10:16:47.00	PDSI	66.6	10:16:26.0
			N	10:16:56.00			
			U	10:06:41.00			
	PKRT	162.26	E	10:16:10.00	SDSI	177.6	10:16:37.6
			N	10:16:10.00			
			U	10:06:44.00			
Simelue 11 April 2012 Mw 8.6	PBLI	483.33	E	8:38:37.00	TPTI	466.2	8:39:37.20
			N	8:39:12.00			
			U	8:40:57.00			
	BITI	545.94	E	8:41:19.00	KCSI	543.9	8:39:47.90
			N	8:39:30.00			
			U	8:54:24.00			
Palu 28 September 2018 Mw 7.5	CMLI	297.62	E	10:04:07.00	SRSI	277.5	10:03:24.9
			N	10:04:00.00			
			U	10:00:50.00			
	CBAL	352.43	E	10:06:56.00	BKB	344.1	10:03:31.2
			N	10:04:39.00			
			U	10:06:53.00			

## Data Availability

Earthquake parameters, including origin time, hypocenter, and moment magnitude (Mw), were sourced from the InaTEWS Earthquake Repository, maintained by the Meteorological, Climatological, and Geophysical Agency of Indonesia (BMKG), accessible at https://repogempa.bmkg.go.id/ (accessed on 3 December 2024).

## Supplementary Materials

The following supporting information can be downloaded at: https://www.mdpi.com/article/10.3390/s25133860/s1, Supplementary Materials for this study include wavelet-based P-wave detection results for the 2009 Padang (Mw 7.6), 2012 Simeulue (Mw 8.6), and 2018 Palu (Mw 7.5) earthquakes, detailed in Figures S1–S3, showing P-wave arrivals at GNSS stations PSKI (69.62 km), PKRT (162.26 km), PBLI (483.33 km), BITI (545.94 km), CMLI (297.62 km), and CBAL (352.43 km), and the wavelet power, thresholds, and spectrograms validated against seismometer stations (PDSI, TPTI, SRSI; Table 1). Figure S4 presents the raw GNSS displacement time series (East, North, Up) alongside the seismometer velocity waveforms, illustrating the input data. Figures S5–S7 compare wavelet-based and STA/LTA P-wave detection for the three earthquakes, demonstrating wavelet robustness with offsets. Text S1 Technical details of the wavelet-based P-wave detection method using CWT with a Mexican hat wavelet, optimized for 1 Hz GNSS data.
