# Supplementary material for: Wavelet-Based P-Wave Detection in High-Rate GNSS Data: A Novel Approach for Rapid Earthquake Monitoring in Tsunamigenic Settings
Source: Sensors (Basel). 2025 Jun 21;25(13):3860. doi: 10.3390/s25133860 (PMC12251905; doi:10.3390/s25133860)
Supplement: Supplementary file 1 [file sensors-25-03860-s001.zip › sensors-3671095-supplementary.pdf]

Supplementary Materials

Figures

Figure S1: Wavelet-Based P-Wave Detection for the 2009 Padang Earthquake (Mw 7.6) at PSKI and PKRT GNSS Stations. (a) East (E), North (N), and Up (U) component waveforms at PSKI (69.62 km from epicenter), with P-wave arrivals at 10:16:47 UTC (E, +21.0 s), 10:16:56 UTC (N, +30.0 s), and 10:06:41 UTC (U, −585.0 s), showing wavelet power (blue), dynamic thresholds (yellow), and spectrograms. (b) E, N, U components at PKRT (76.42 km), with arrivals at 10:16:08.4 UTC (E/N, −27.6 s) and 10:06:44.0 UTC (U, −593.6 s). Data are validated against BMKG seismic records (Table 1).

S1 (a) PSKI GNSS Station

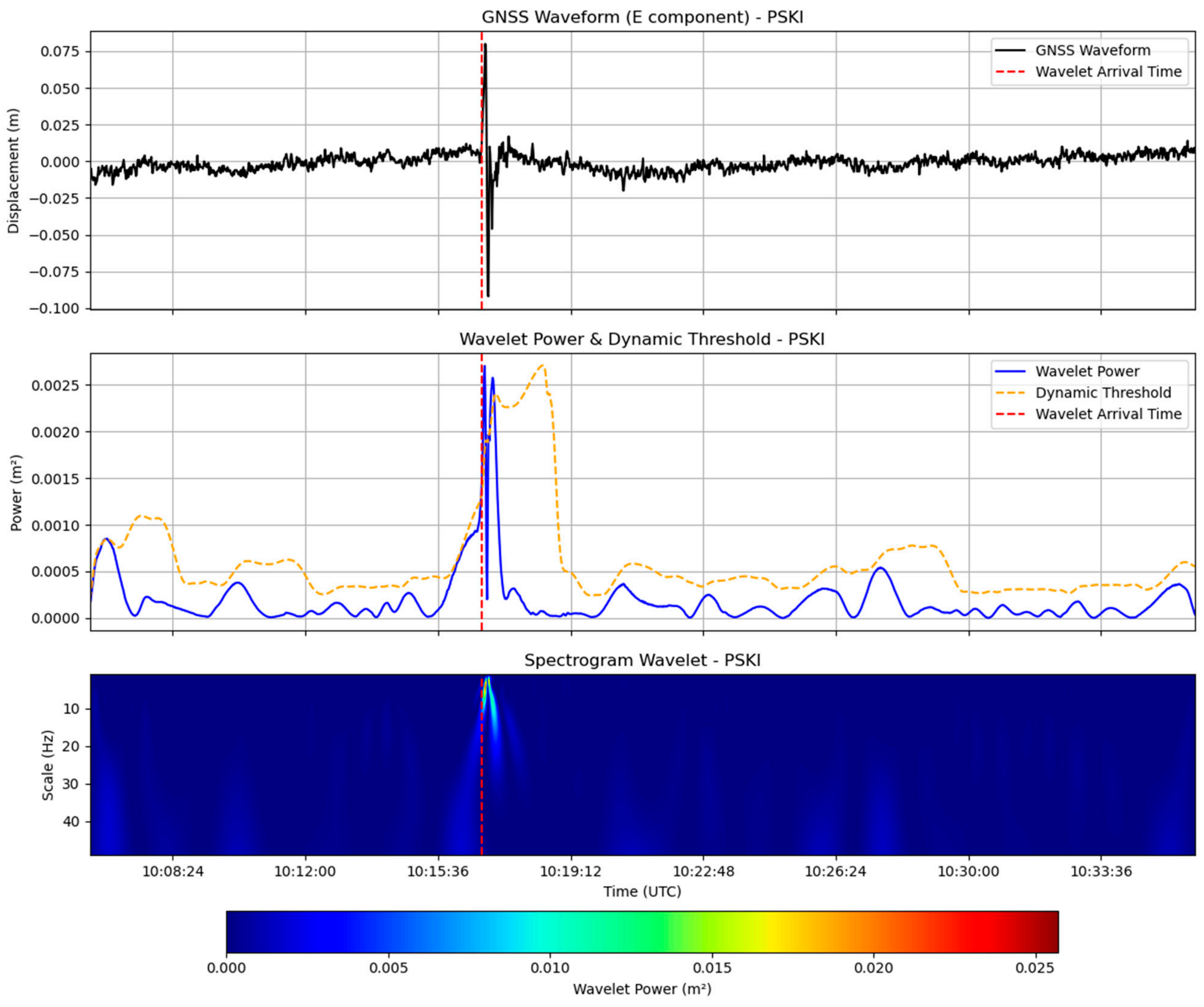

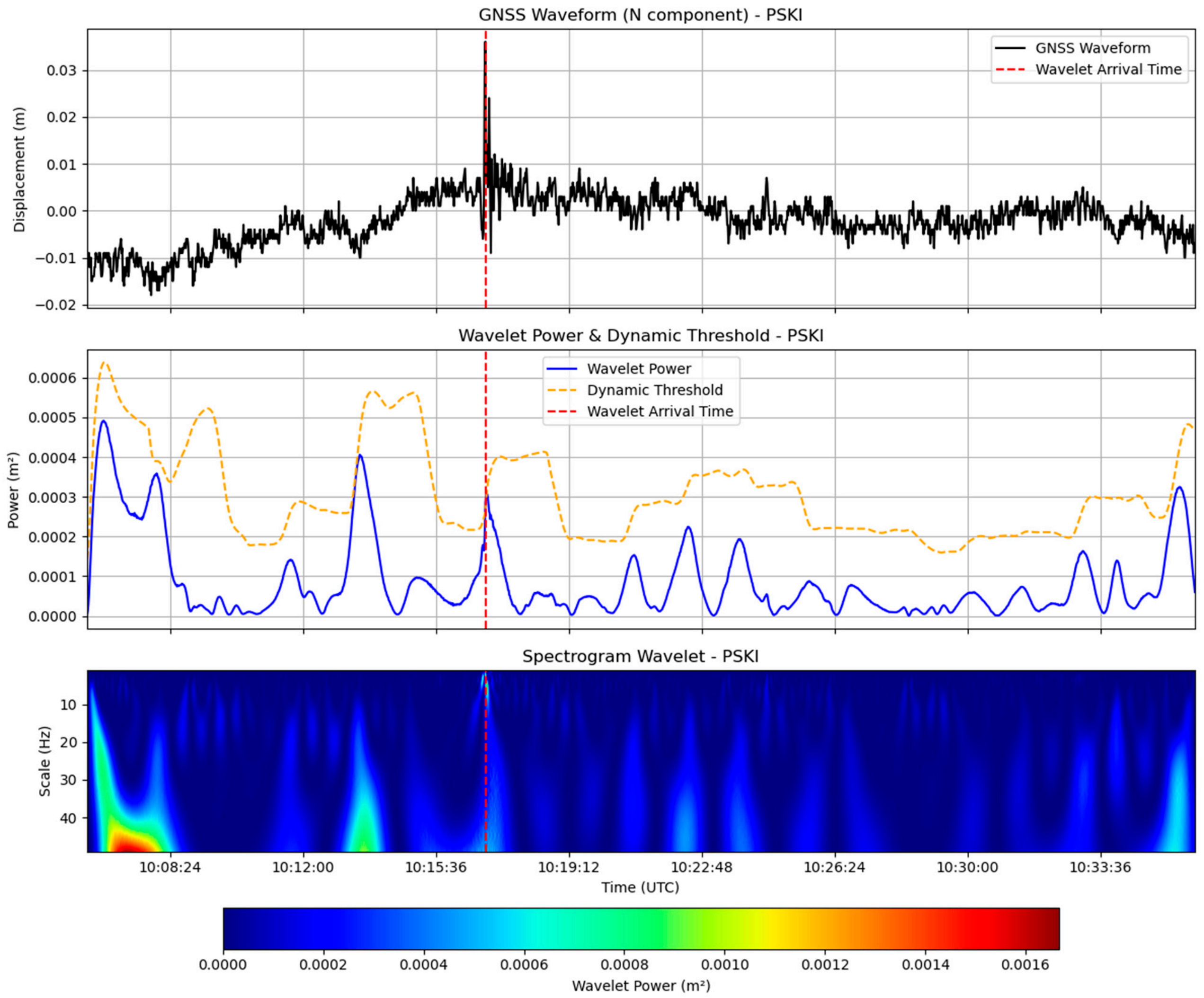
GNSS Waveform (N component) - PSKI
Displacement (m)
GNSS Waveform
Wavelet Arrival Time
Wavelet Power & Dynamic Threshold - PSKI
Power (m²)
Wavelet Power
Dynamic Threshold
Wavelet Arrival Time
Spectrogram Wavelet - PSKI
Scale (Hz)
10:08:24
10:12:00
10:15:36
10:19:12
10:22:48
10:26:24
10:30:00
10:33:36
Time (UTC)
Wavelet Power (m²)

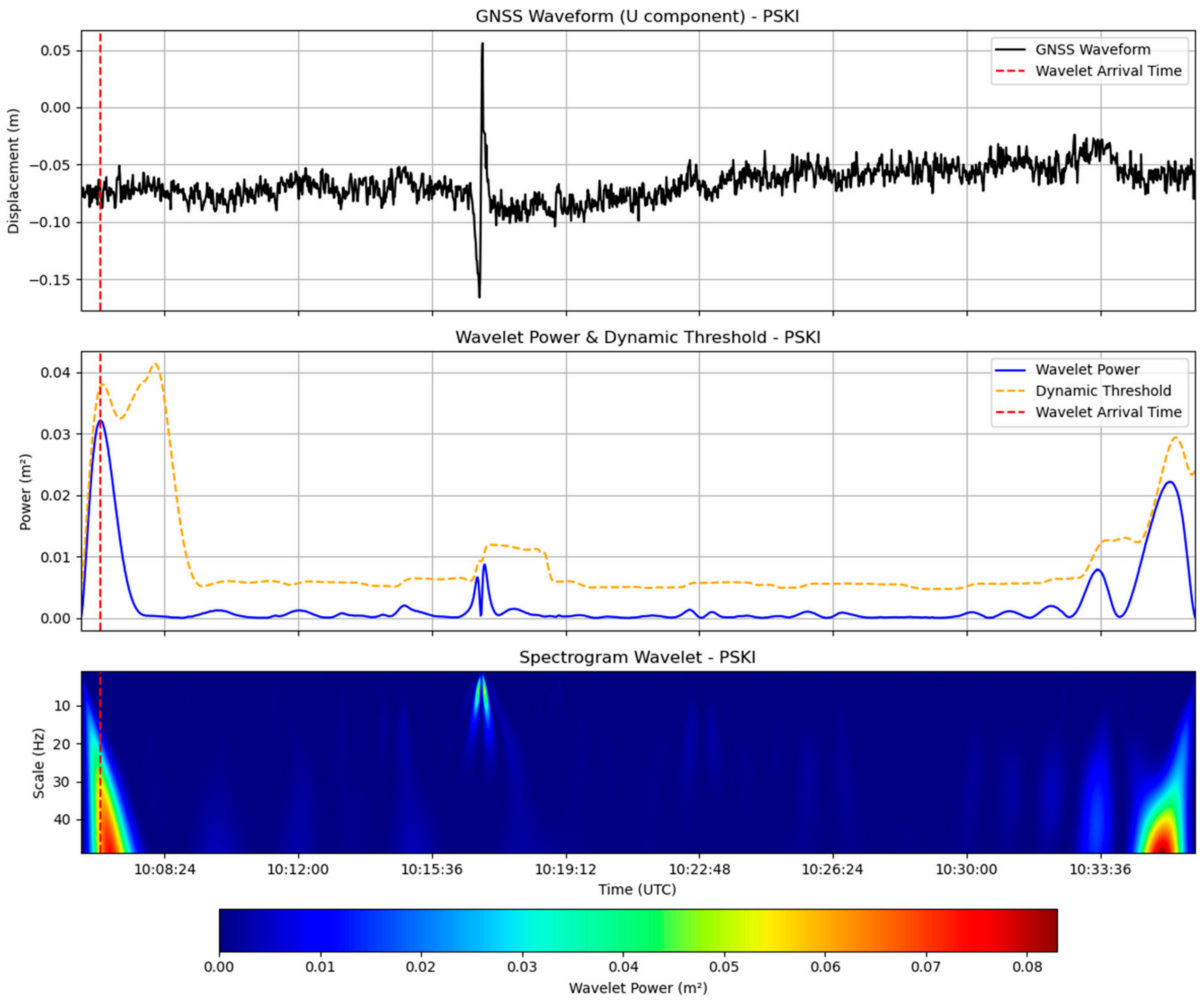
GNSS Waveform (U component) - PSKI
Displacement (m)
GNSS Waveform
Wavelet Arrival Time
Wavelet Power & Dynamic Threshold - PSKI
Power (m²)
Wavelet Power
Dynamic Threshold
Wavelet Arrival Time
Spectrogram Wavelet - PSKI
Scale (Hz)
10:08:24
10:12:00
10:15:36
10:19:12
10:22:48
10:26:24
10:30:00
10:33:36
Time (UTC)
Wavelet Power (m²)

S1 (b) PKRT GNSS Station

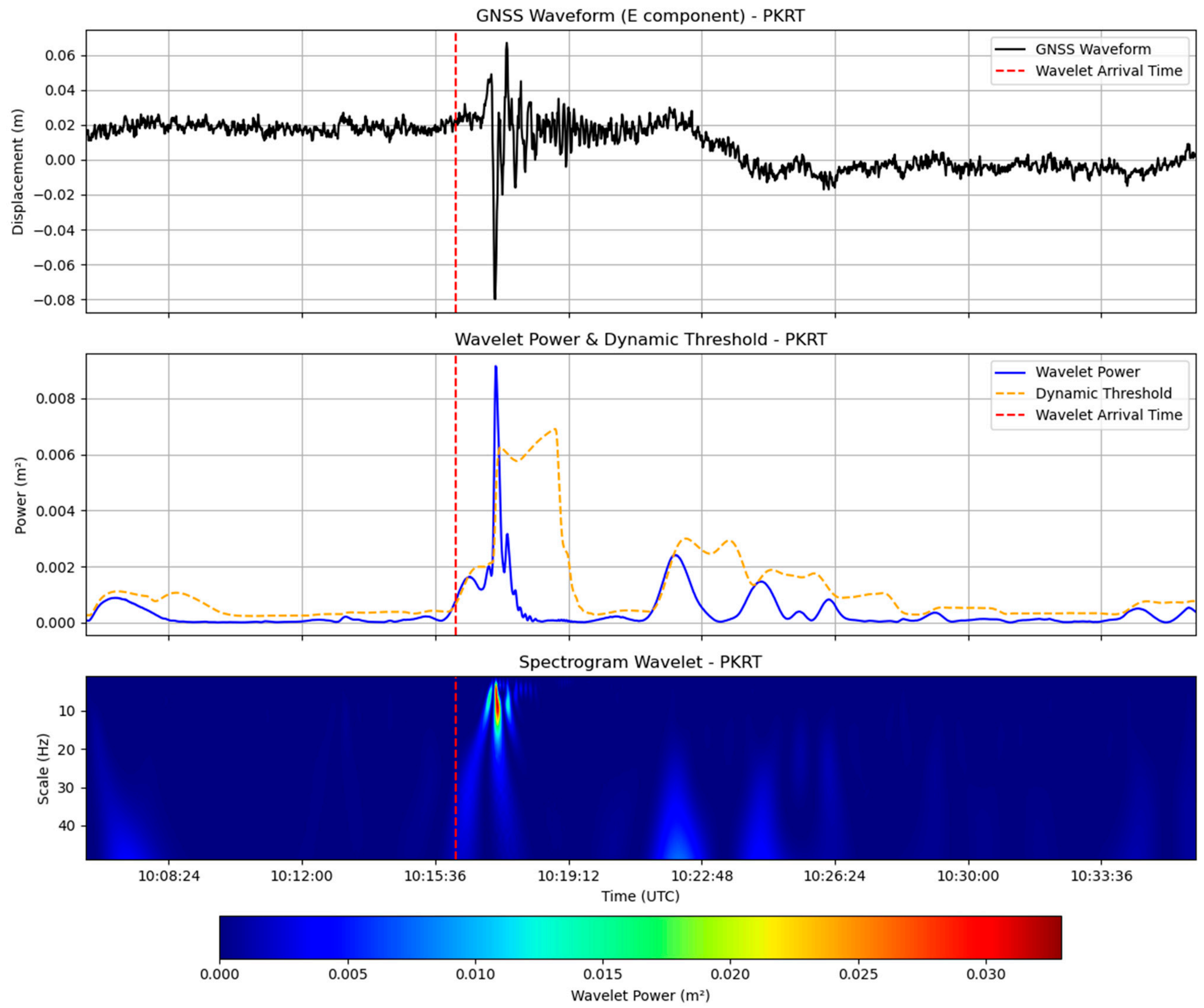

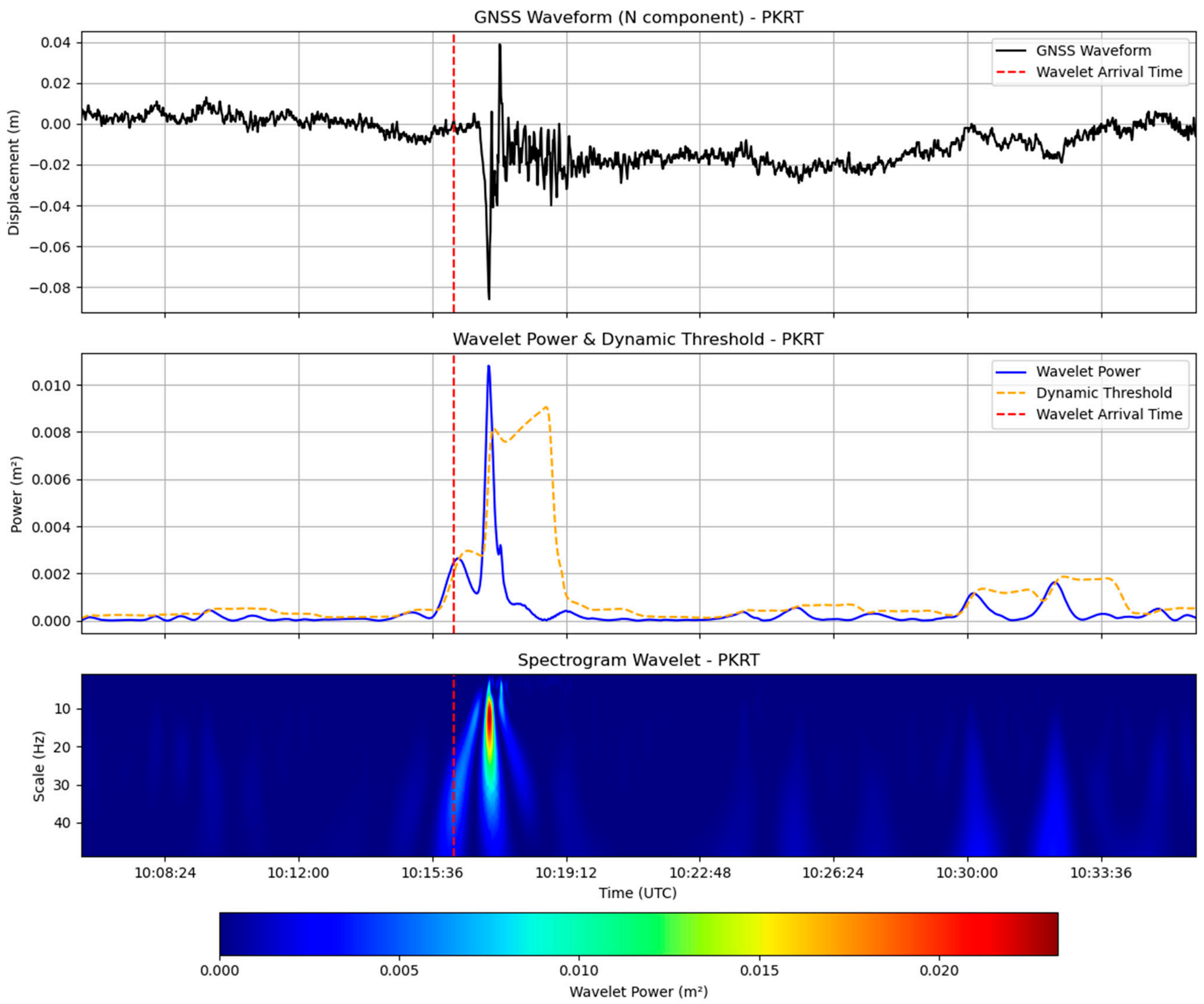

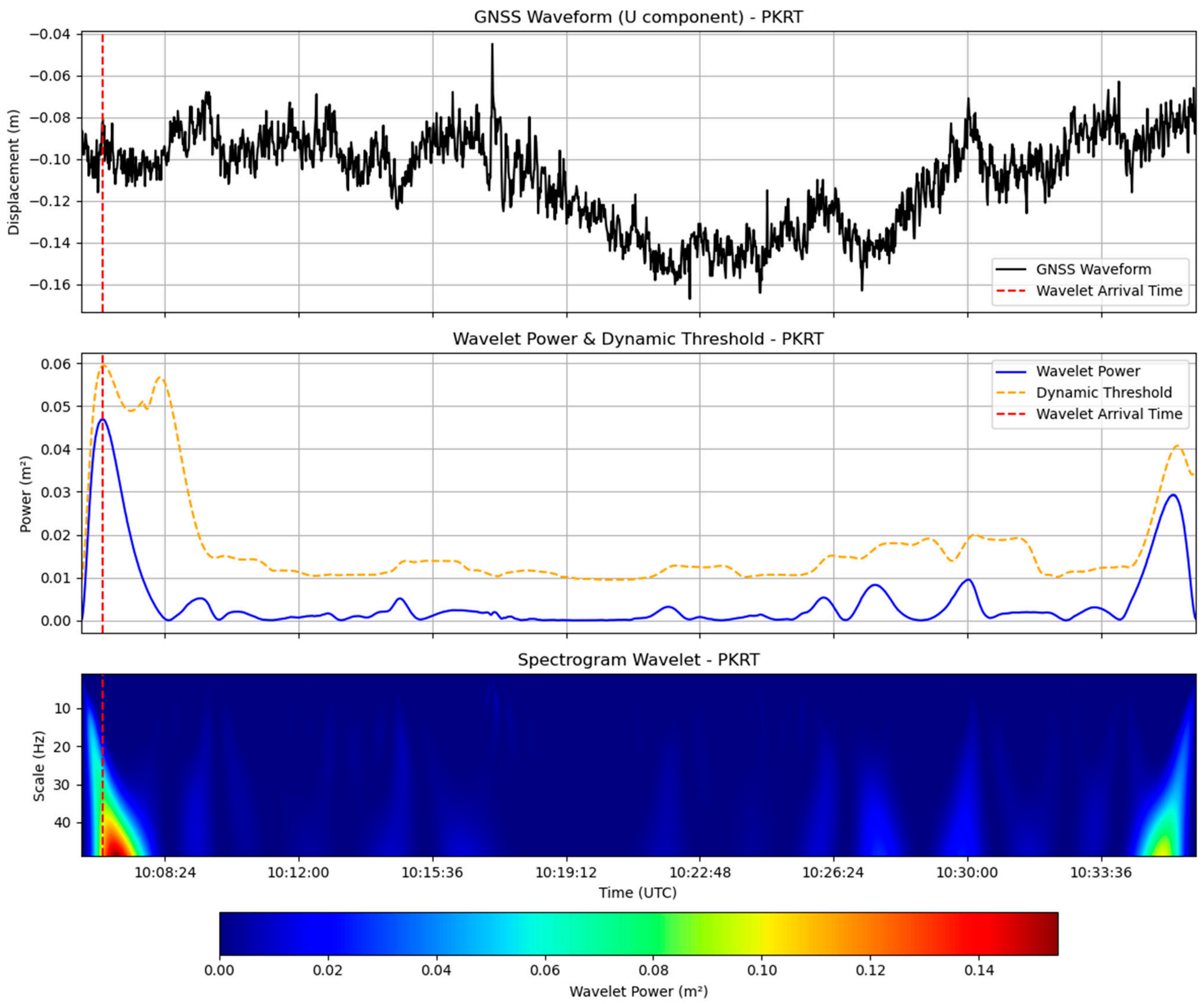
GNSS Waveform (U component) - PKRT
Displacement (m)
GNSS Waveform
Wavelet Arrival Time
Wavelet Power & Dynamic Threshold - PKRT
Power (m²)
Wavelet Power
Dynamic Threshold
Wavelet Arrival Time
Spectrogram Wavelet - PKRT
Scale (Hz)
10:08:24
10:12:00
10:15:36
10:19:12
10:22:48
10:26:24
10:30:00
10:33:36
Time (UTC)
Wavelet Power (m²)

Figure S2: Wavelet-Based P-Wave Detection for the 2012 Simeulue Earthquake (Mw 8.6) at PBLI and BITI GNSS Stations. (a) E, N, U components at PBLI (483.33 km), with arrivals at 08:38:37 UTC (E, -60.2 s), 08:39:12 UTC (N, -25.2 s), and 08:40:57 UTC (U, 79.8 s). (b) E, N, U components at BITI (545.94 km), with arrivals at 08:41:19 UTC (E, +91.1 s), 08:39:30 UTC (N, -17.9 s), and 08:54:24 UTC (U, +876.1 s). Data are validated against BMKG seismic records (Table 1).

S2 (a) PBLI GNSS Station

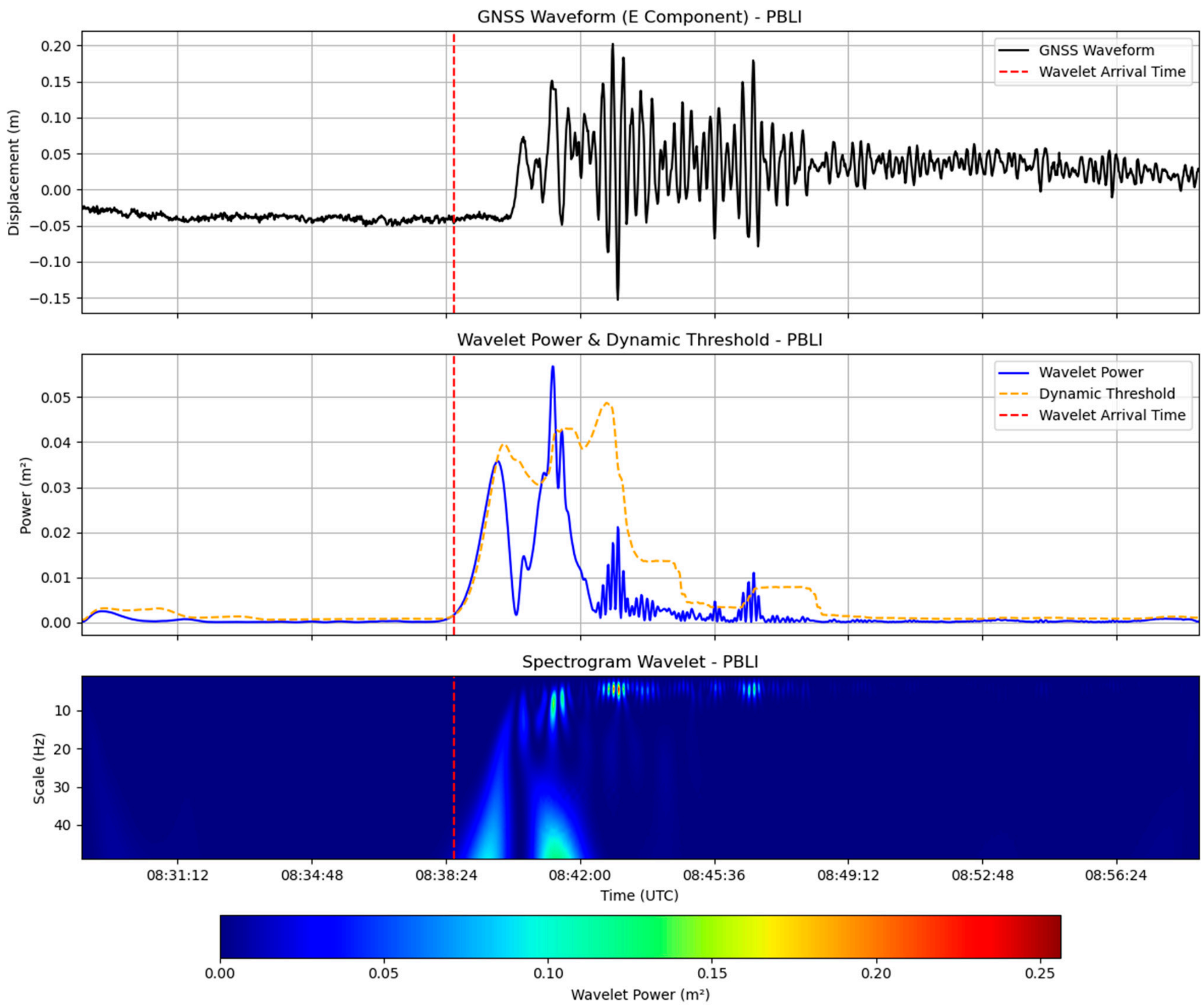

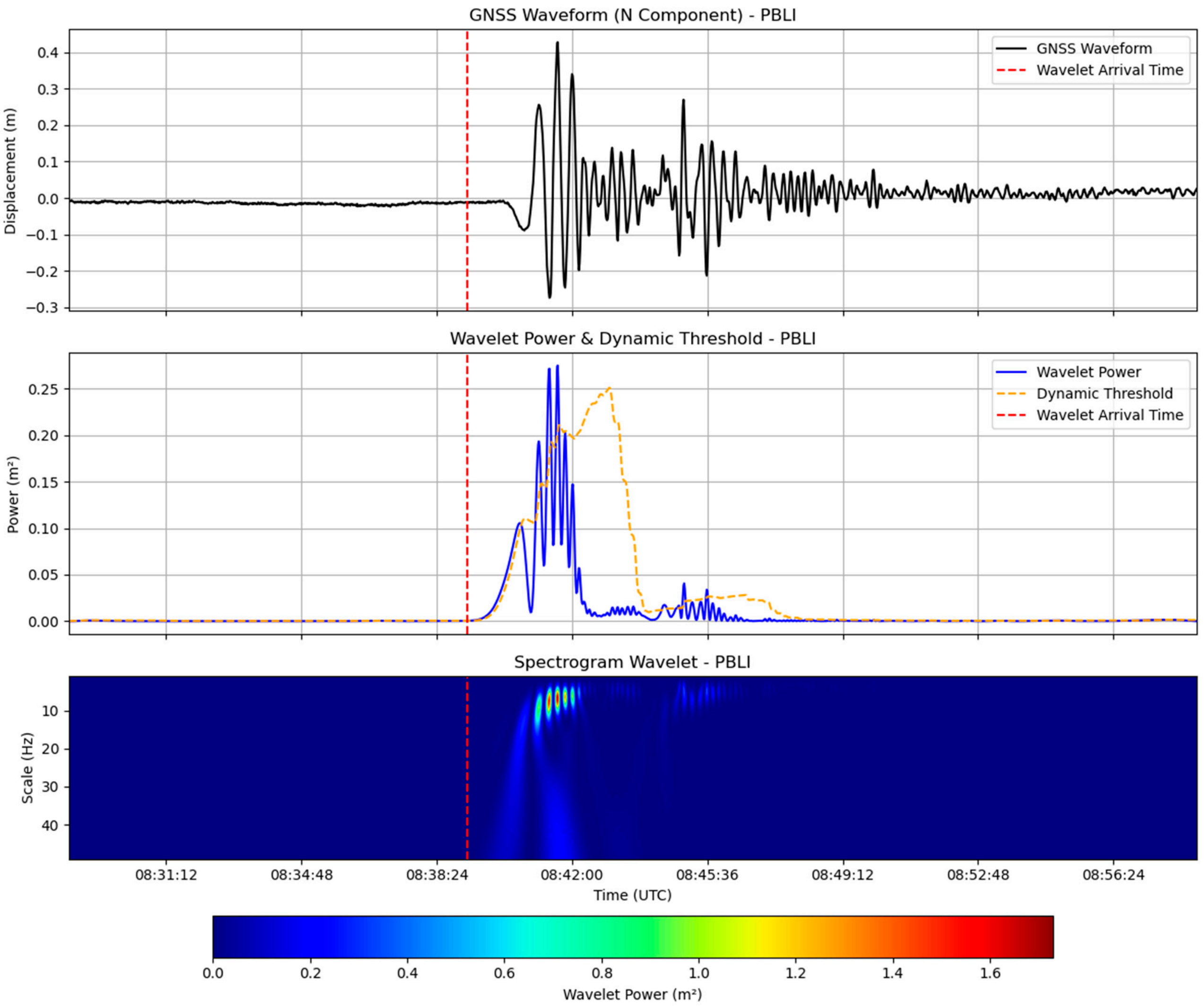
GNSS Waveform (N Component) - PBLI
Displacement (m)
GNSS Waveform
Wavelet Arrival Time
0.4
0.3
0.2
0.1
0.0
−0.1
−0.2
−0.3
Wavelet Power & Dynamic Threshold - PBLI
Power (m²)
Wavelet Power
Dynamic Threshold
Wavelet Arrival Time
0.25
0.20
0.15
0.10
0.05
0.00
Spectrogram Wavelet - PBLI
Scale (Hz)
10
20
30
40
08:31:12
08:34:48
08:38:24
08:42:00
08:45:36
08:49:12
08:52:48
08:56:24
Time (UTC)
0.0
0.2
0.4
0.6
0.8
1.0
1.2
1.4
1.6
Wavelet Power (m²)

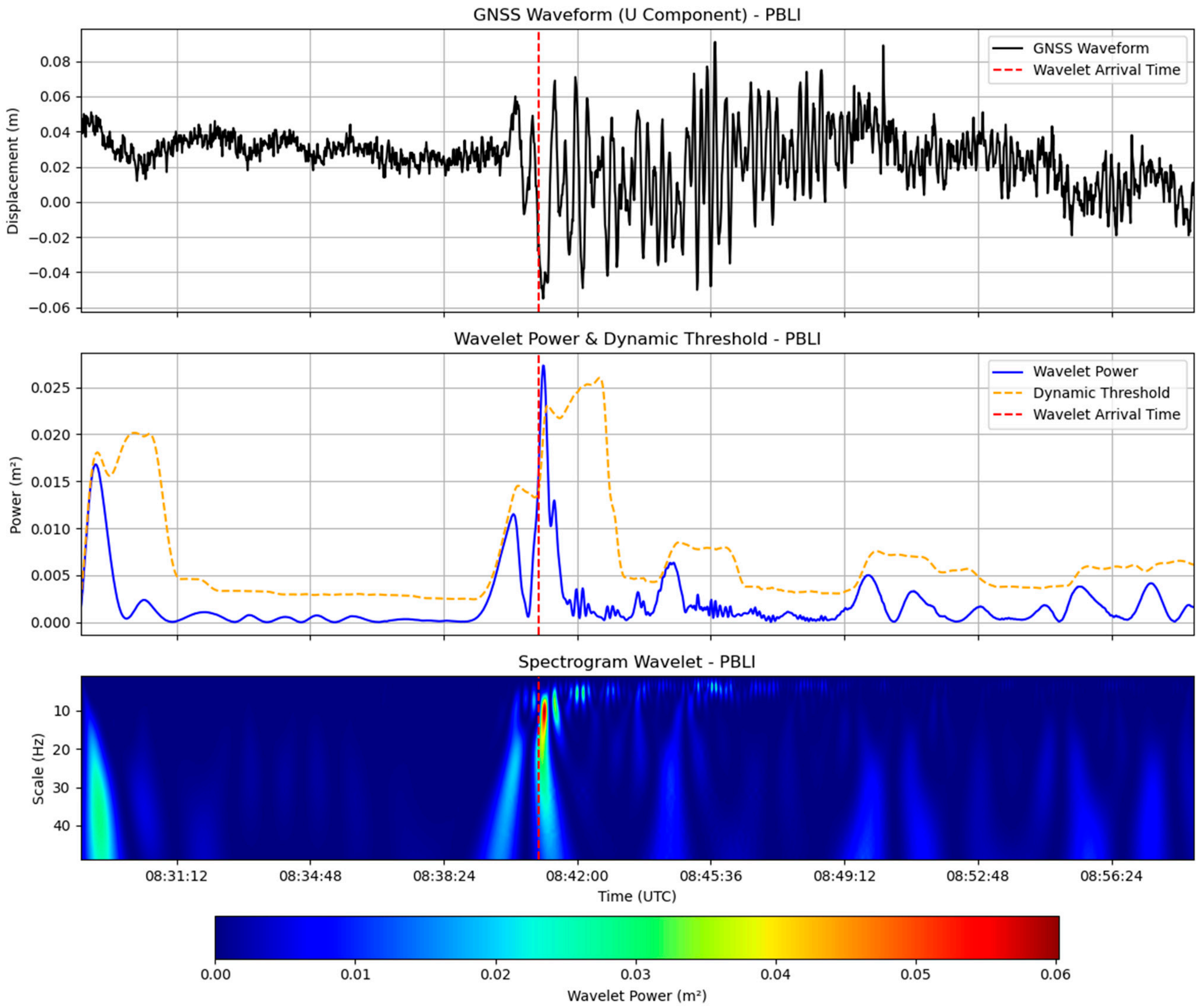
GNSS Waveform (U Component) - PBLI
Displacement (m)
GNSS Waveform
Wavelet Arrival Time
0.08
0.06
0.04
0.02
0.00
−0.02
−0.04
−0.06
Wavelet Power & Dynamic Threshold - PBLI
Power (m²)
Wavelet Power
Dynamic Threshold
Wavelet Arrival Time
0.025
0.020
0.015
0.010
0.005
0.000
Spectrogram Wavelet - PBLI
Scale (Hz)
10
20
30
40
08:31:12
08:34:48
08:38:24
08:42:00
08:45:36
08:49:12
08:52:48
08:56:24
Time (UTC)
0.00
0.01
0.02
0.03
0.04
0.05
0.06
Wavelet Power (m²)

S2 (b) BITI GNSS Station

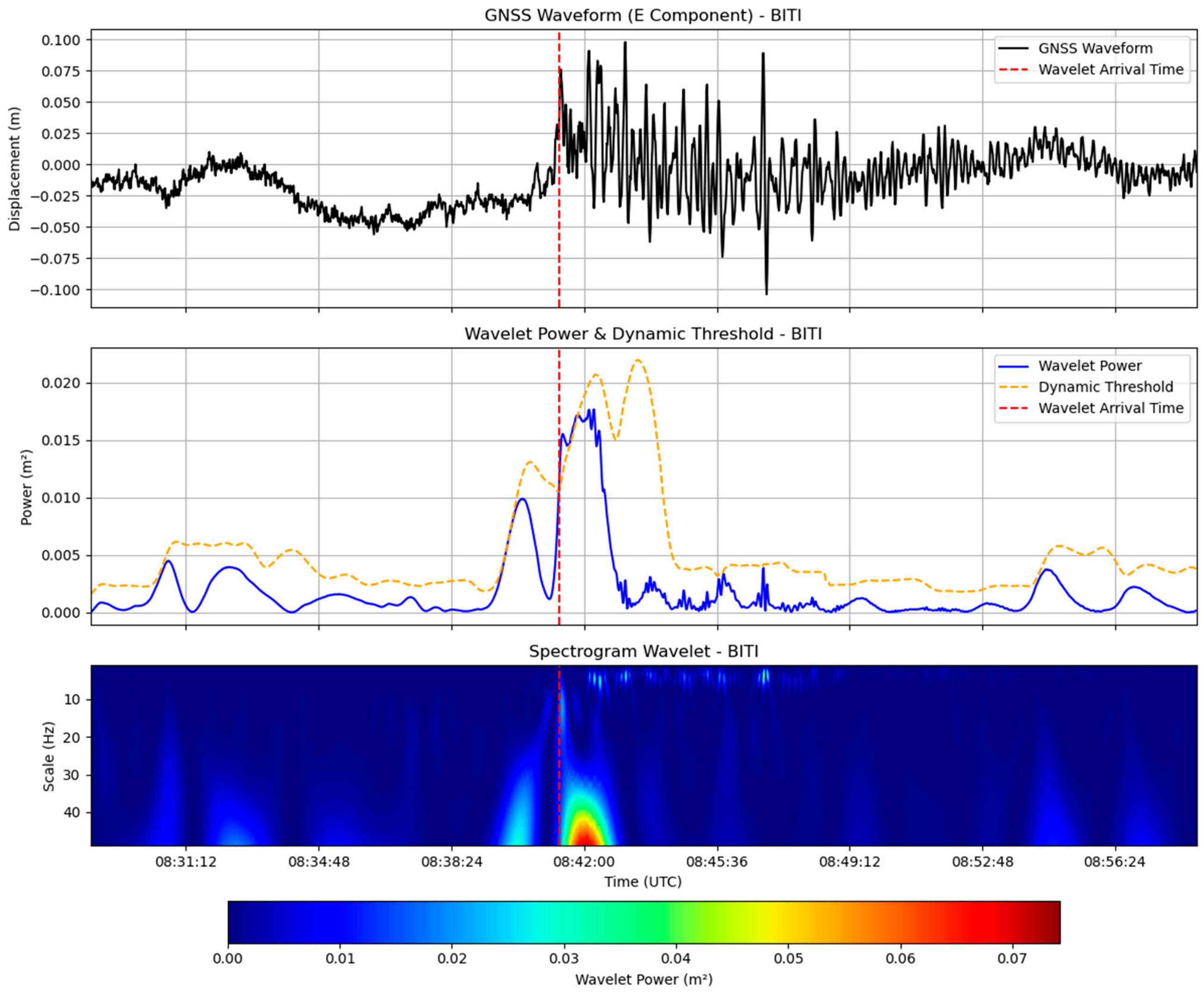

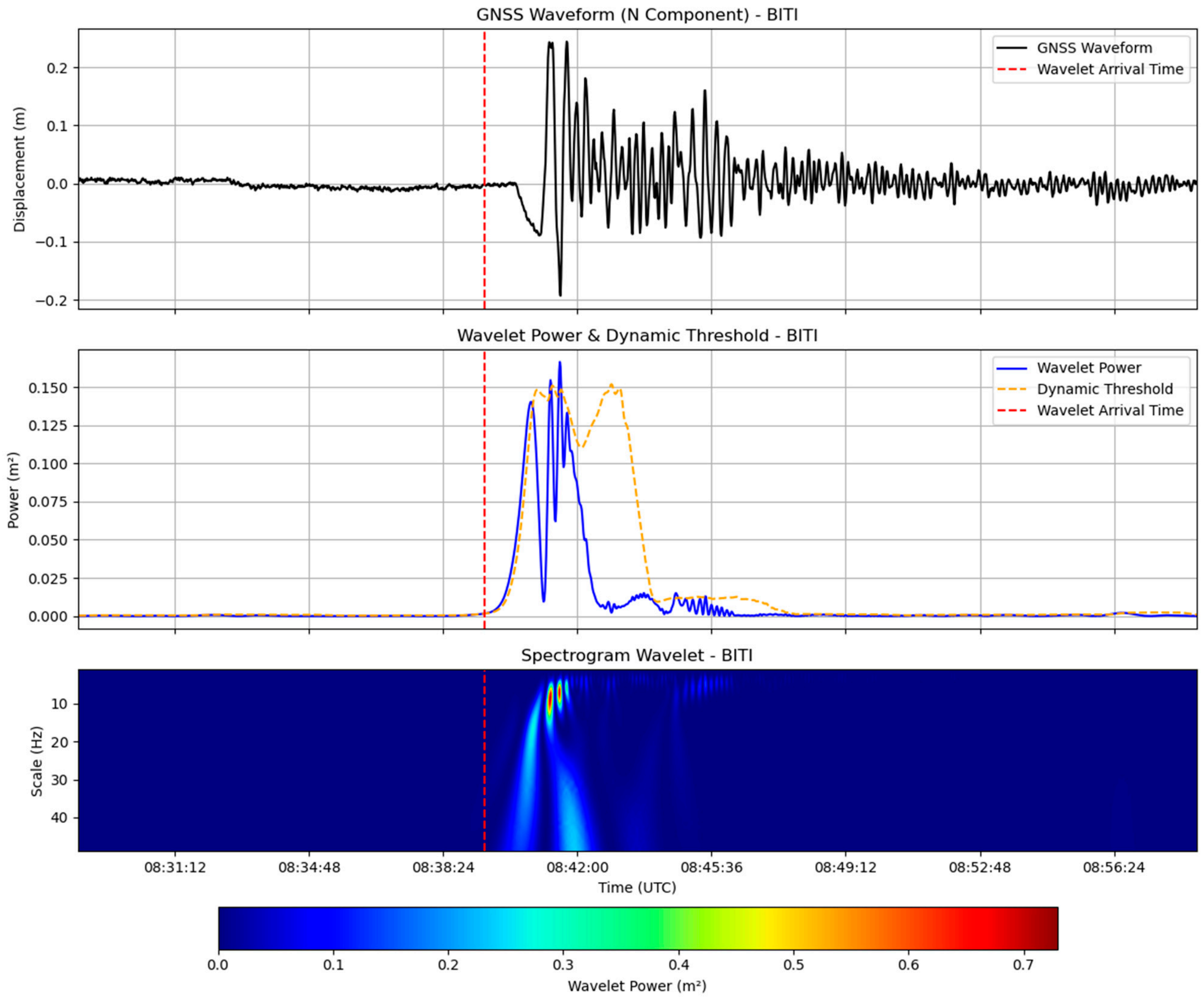

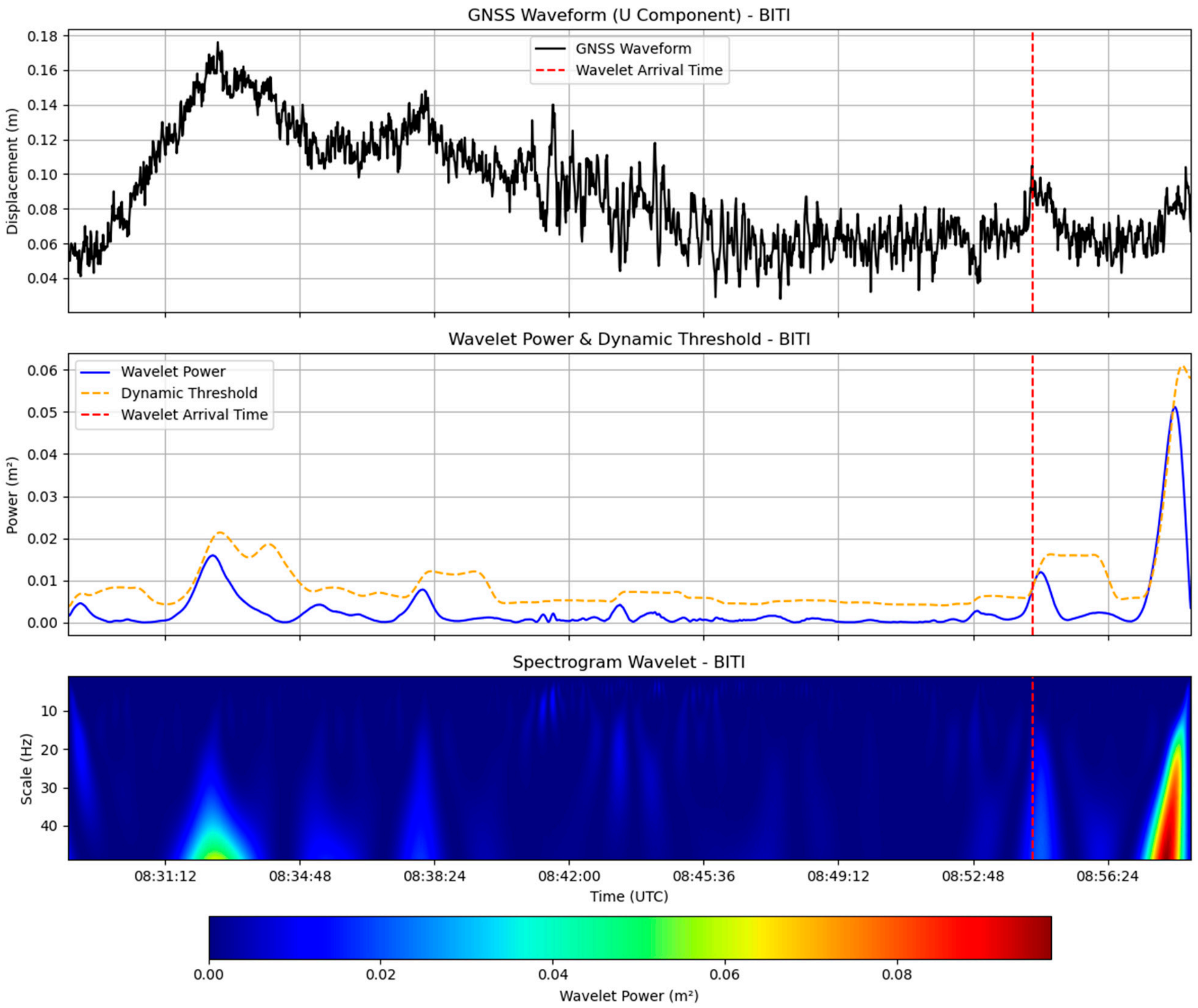

GNSS Waveform (U Component) - BITI
GNSS Waveform
Wavelet Arrival Time
Displacement (m)
0.18
0.16
0.14
0.12
0.10
0.08
0.06
0.04
Wavelet Power & Dynamic Threshold - BITI
Wavelet Power
Dynamic Threshold
Wavelet Arrival Time
Power (m²)
0.06
0.05
0.04
0.03
0.02
0.01
0.00
Spectrogram Wavelet - BITI
Scale (Hz)
10
20
30
40
08:31:12
08:34:48
08:38:24
08:42:00
08:45:36
08:49:12
08:52:48
08:56:24
Time (UTC)
0.00
0.02
0.04
0.06
0.08
Wavelet Power (m²)

Figure S3: Wavelet-Based P-Wave Detection for the 2018 Palu Earthquake (Mw 7.5) at CMLI and CBAL GNSS Stations. (a) E, N, U components at CMLI (297.62 km), with arrivals at 10:04:07 UTC (E, +42.1 s), 10:04:00 UTC (N, +35.1 s), and 10:00:50 UTC (U, -154.9 s). (b) E, N, U components at CBAL (352.43 km), with arrivals at 10:06:56 UTC (E, +204.8 s), 10:04:39 UTC (N, +67.8 s), and 10:06:53 UTC (U, +201.8 s). Data are validated against BMKG seismic records (Table 1).

S3 (a) CMLI GNSS Station

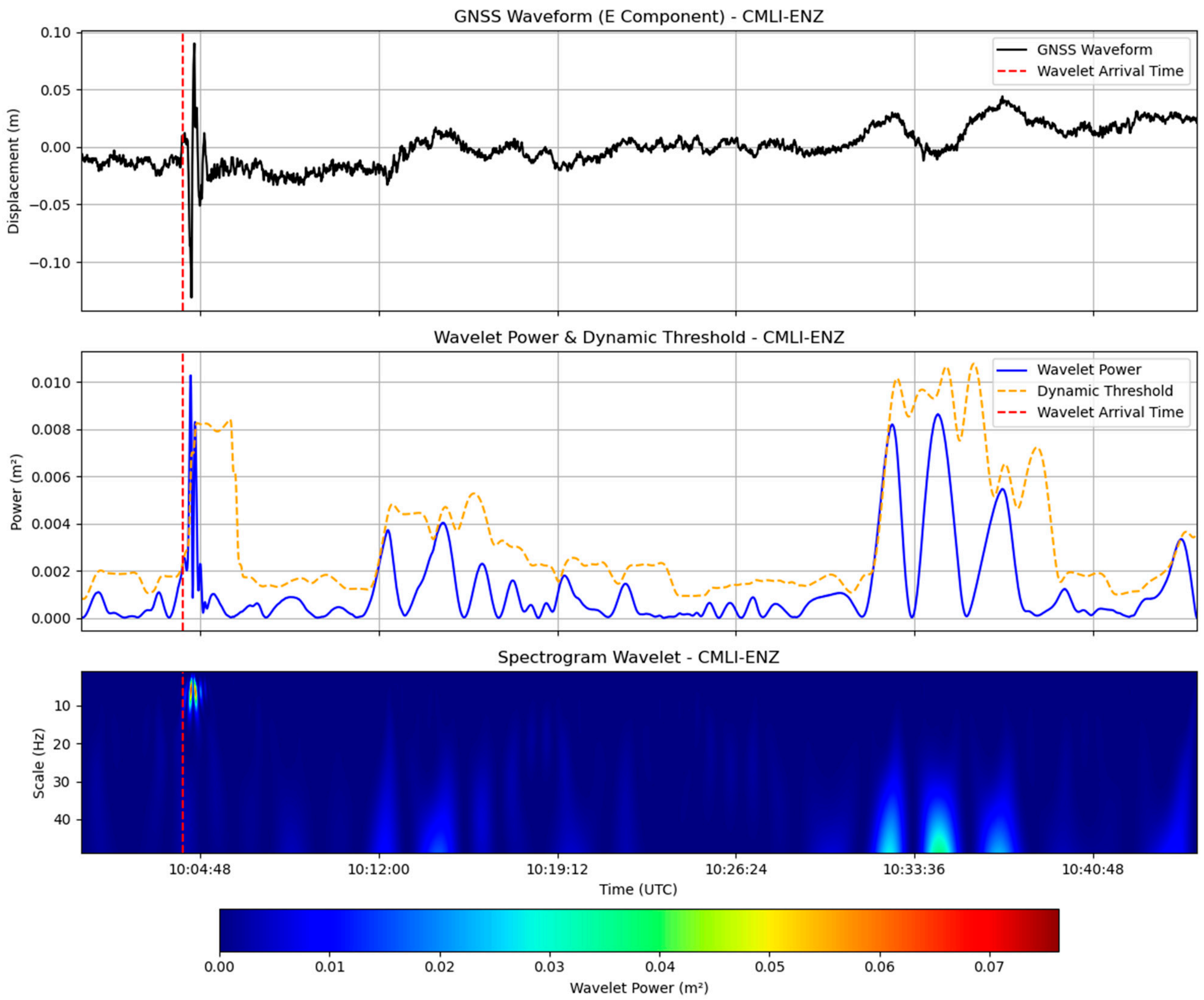

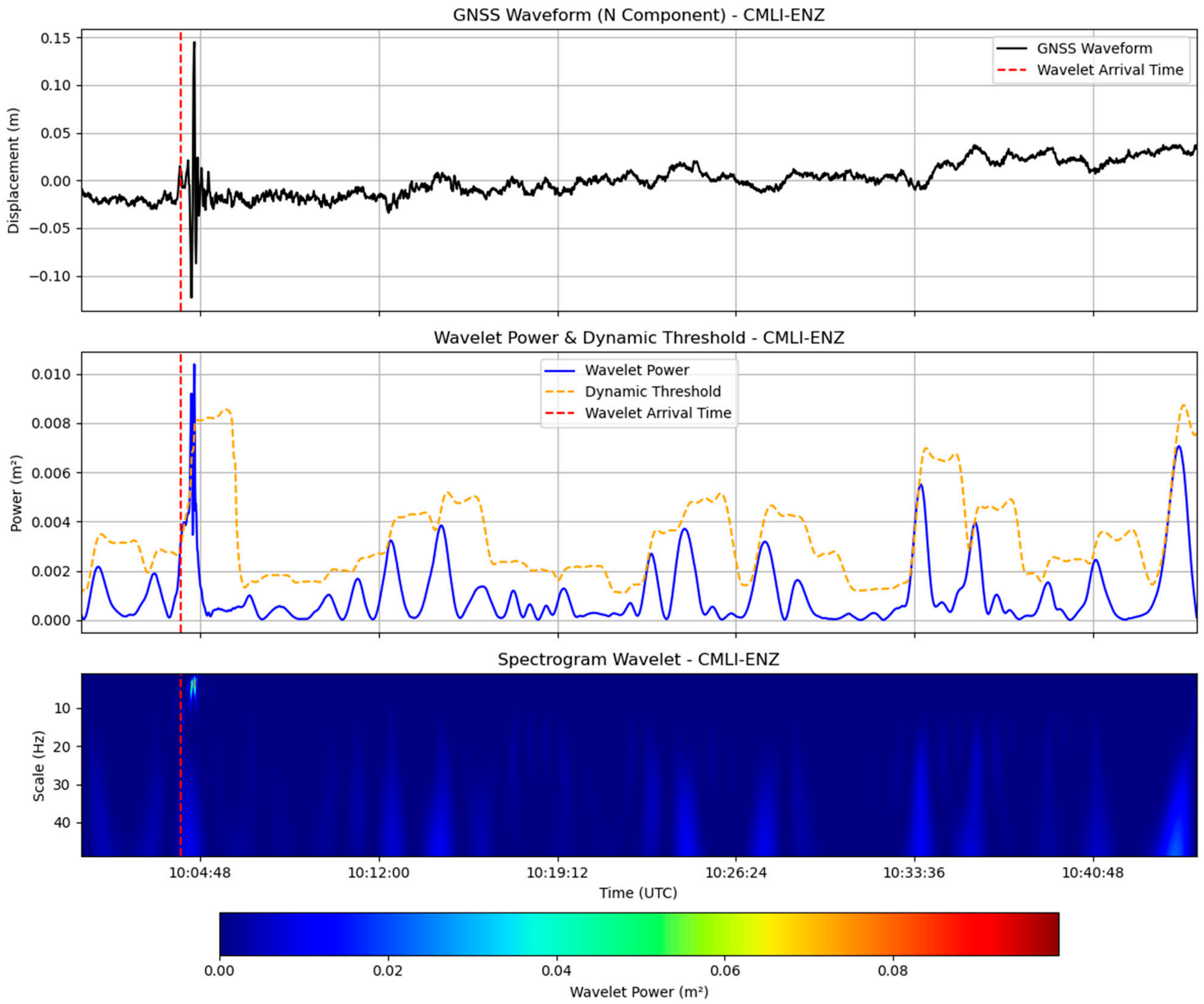
GNSS Waveform (N Component) - CMLI-ENZ
Displacement (m)
GNSS Waveform
Wavelet Arrival Time
Wavelet Power & Dynamic Threshold - CMLI-ENZ
Power (m²)
Wavelet Power
Dynamic Threshold
Wavelet Arrival Time
Spectrogram Wavelet - CMLI-ENZ
Scale (Hz)
10:04:48
10:12:00
10:19:12
10:26:24
10:33:36
10:40:48
Time (UTC)
Wavelet Power (m²)

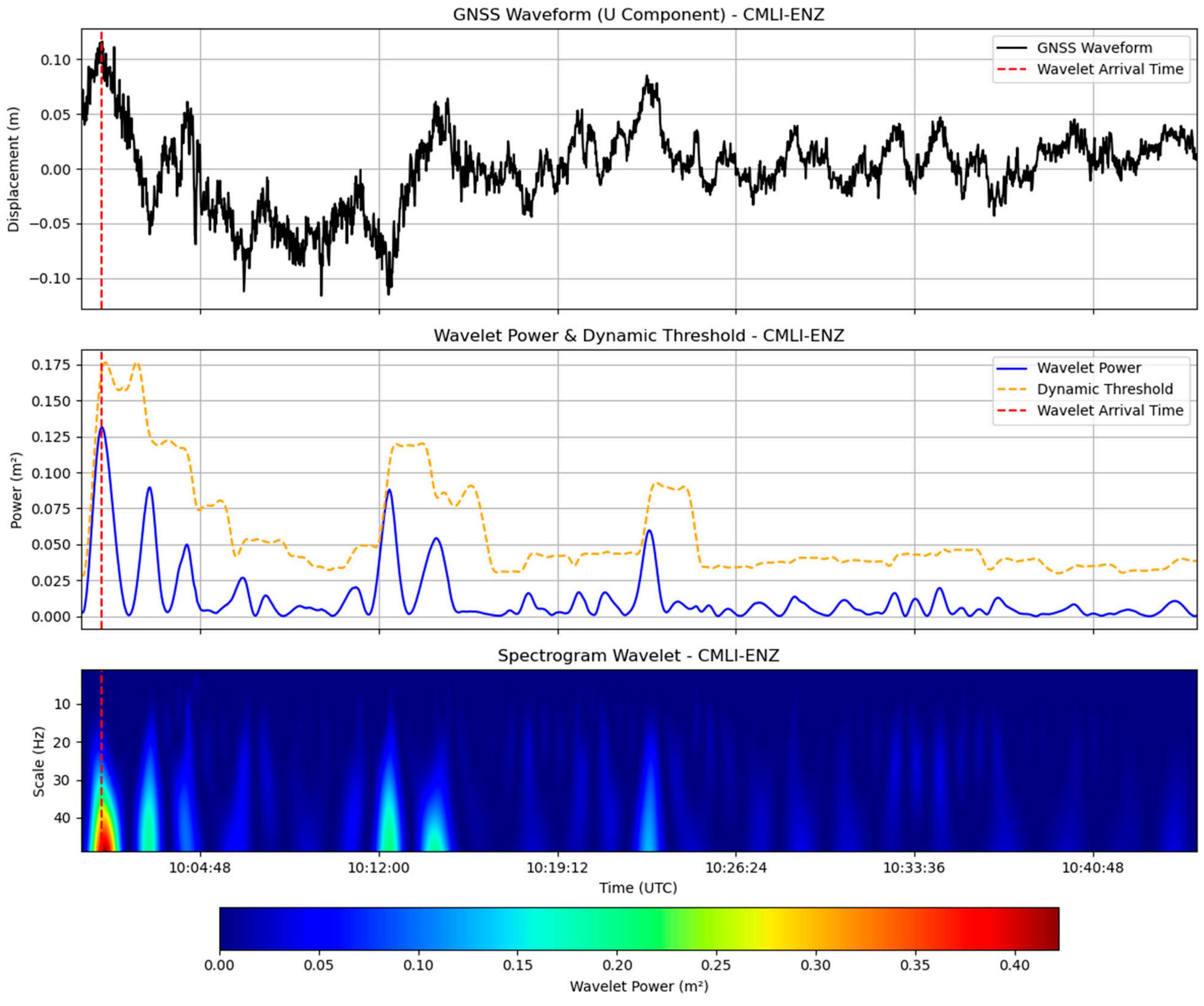
GNSS Waveform (U Component) - CMLI-ENZ
Displacement (m)
GNSS Waveform
Wavelet Arrival Time
Wavelet Power & Dynamic Threshold - CMLI-ENZ
Power (m²)
Wavelet Power
Dynamic Threshold
Wavelet Arrival Time
Spectrogram Wavelet - CMLI-ENZ
Scale (Hz)
10:04:48
10:12:00
10:19:12
10:26:24
10:33:36
10:40:48
Time (UTC)
Wavelet Power (m²)

S3 (b) CBAL GNSS Station

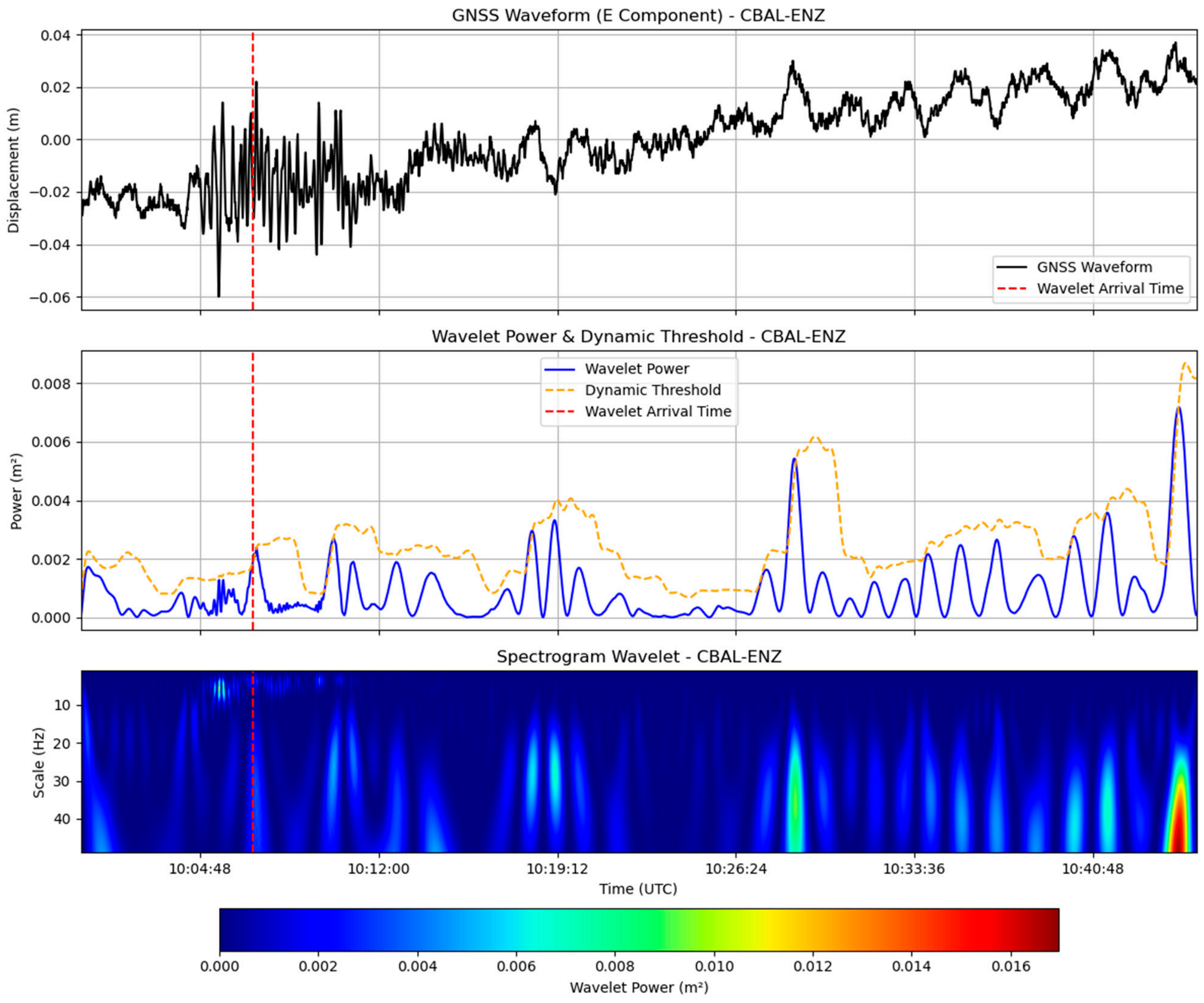

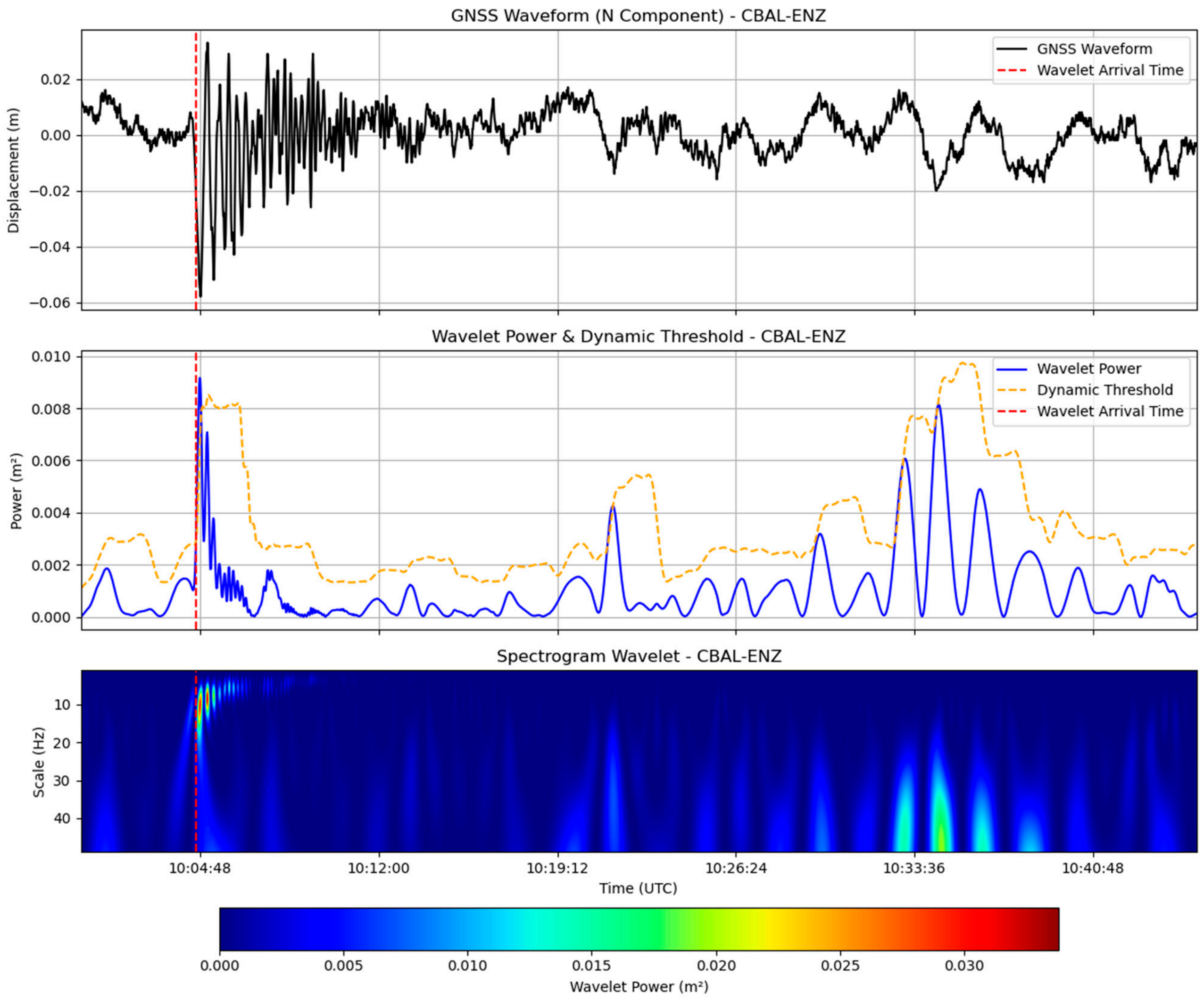

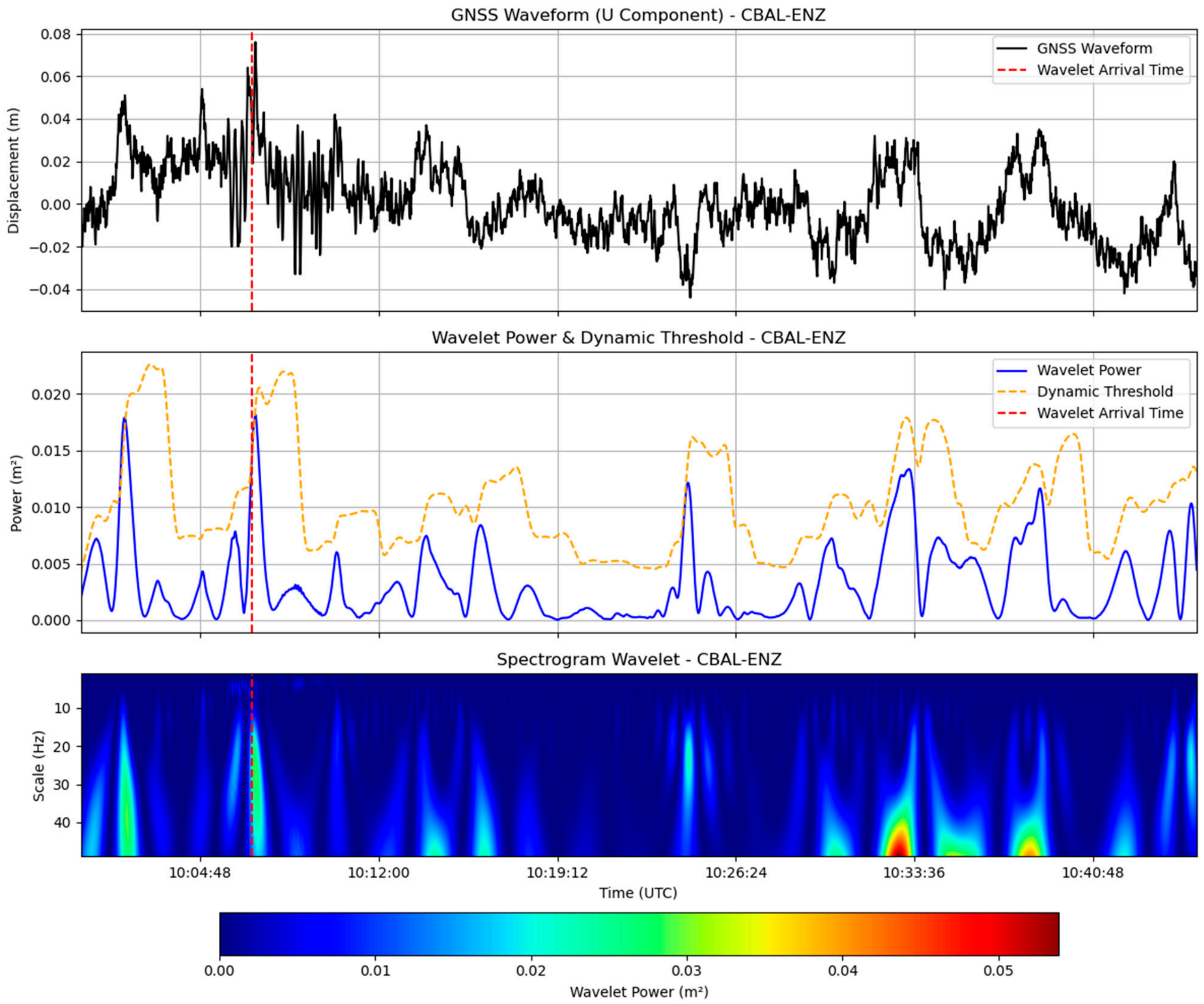
GNSS Waveform (U Component) - CBAL-ENZ
Displacement (m)
0.08
0.06
0.04
0.02
0.00
−0.02
−0.04
GNSS Waveform
Wavelet Arrival Time
Wavelet Power & Dynamic Threshold - CBAL-ENZ
Power (m²)
0.020
0.015
0.010
0.005
0.000
Wavelet Power
Dynamic Threshold
Wavelet Arrival Time
Spectrogram Wavelet - CBAL-ENZ
Scale (Hz)
10
20
30
40
10:04:48
10:12:00
10:19:12
10:26:24
10:33:36
10:40:48
Time (UTC)
0.00
0.01
0.02
0.03
0.04
0.05
Wavelet Power (m²)

Figure S4: Raw GNSS and Seismometer Data for P-Wave Detection Validation. (a) Raw GNSS displacement time series (East, North, Up components) for the 2009 Padang earthquake at PSKI (69.62 km from epicenter), alongside seismometer velocity waveforms at PDSI (66.6 km). (b) Raw GNSS data for the 2012 Simeulue earthquake at PBLI (483.33 km), with seismometer data at KCSI (543.9 km). (c) Raw GNSS data for the 2018 Palu earthquake at CMLI (297.62 km), with seismometer data at BKB (344.1 km). These visualizations illustrate the complementary nature of GNSS absolute displacements and seismometer velocity records, supporting the validation of P-wave detection results (Table 1).

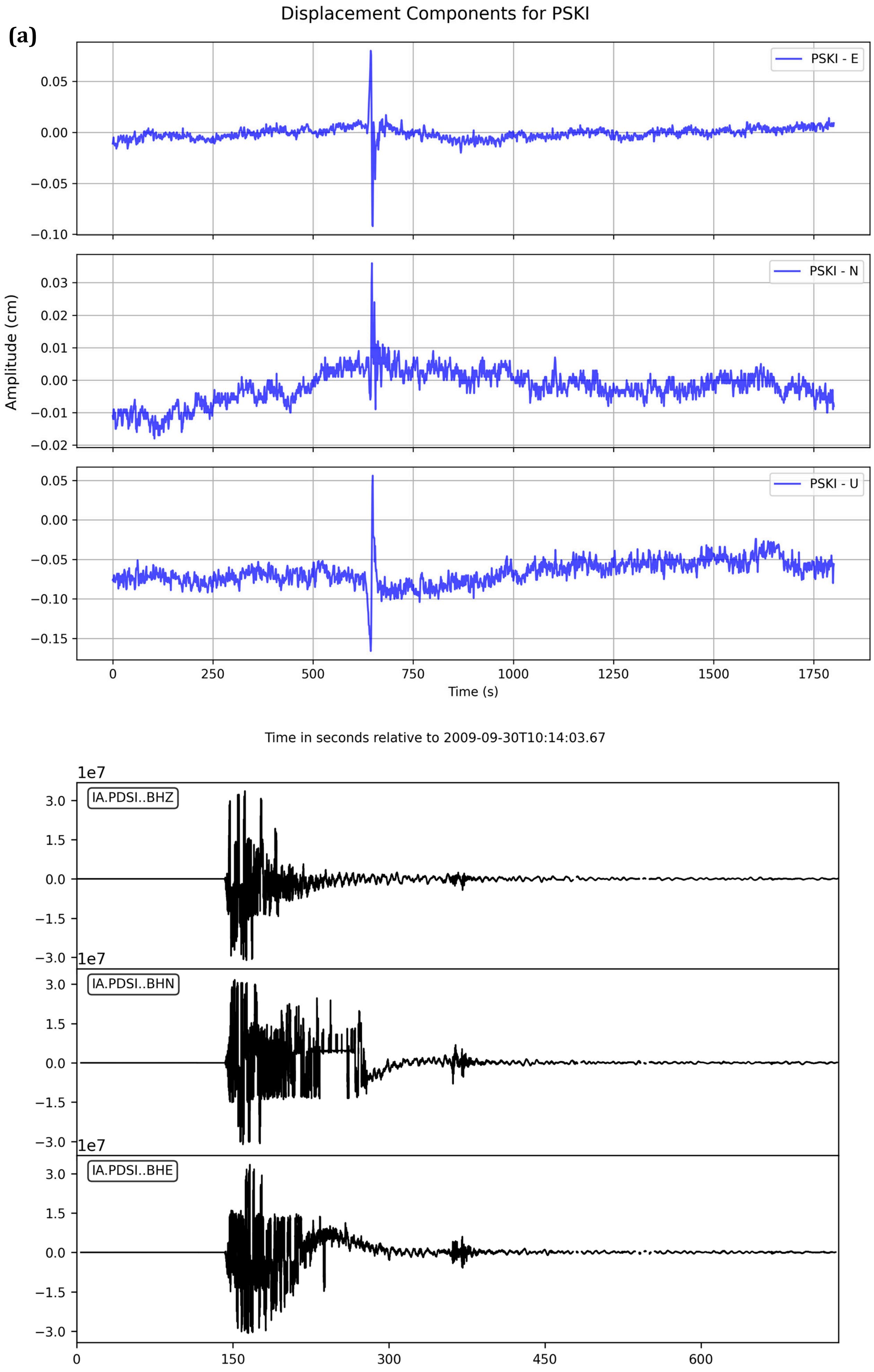

(b)

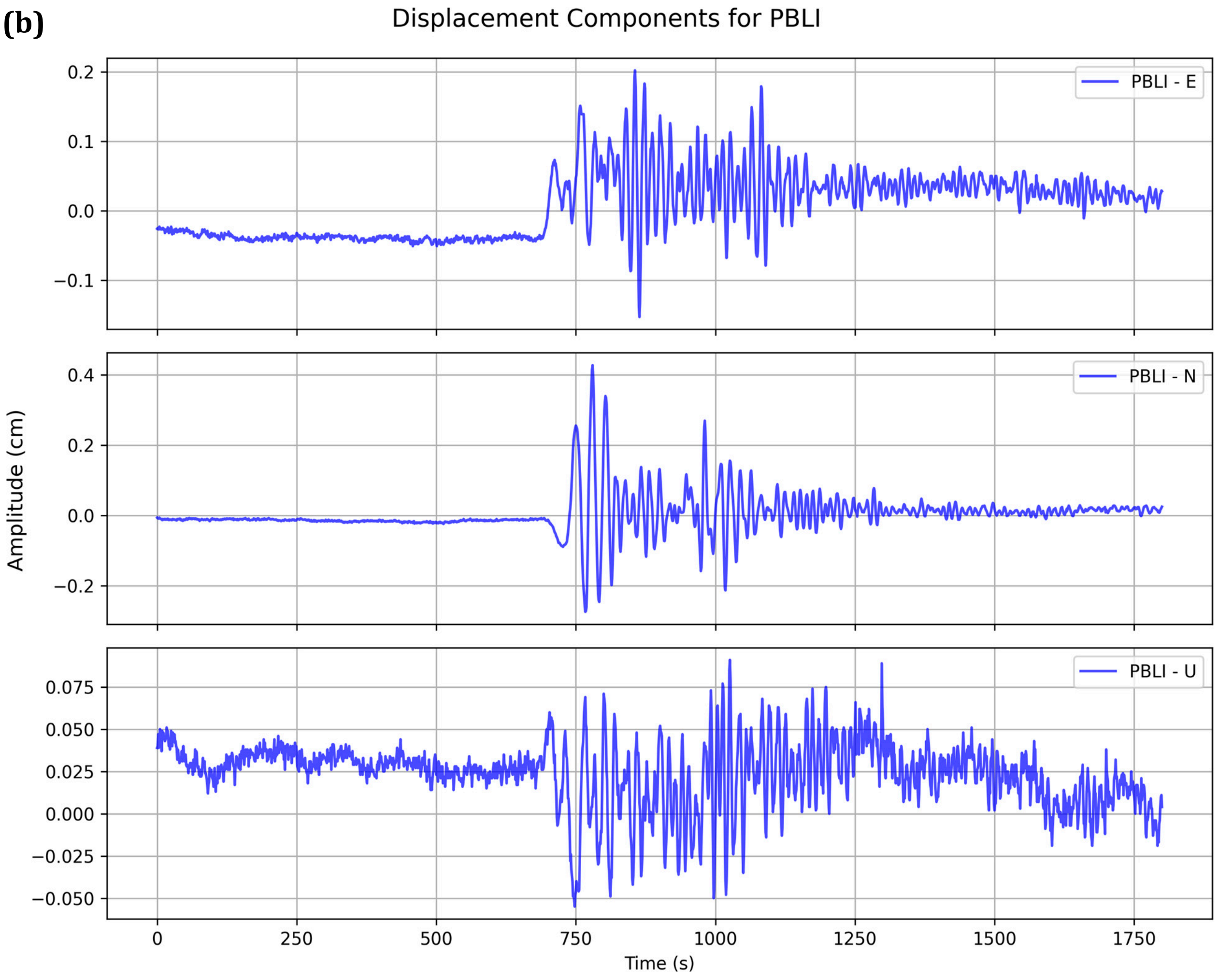
Displacement Components for PBLI
PBLI - E
PBLI - N
PBLI - U
Amplitude (cm)
Time (s)
0.2
0.1
0.0
−0.1
0.4
0.2
0.0
−0.2
0.075
0.050
0.025
0.000
−0.025
−0.050
0
250
500
750
1000
1250
1500
1750

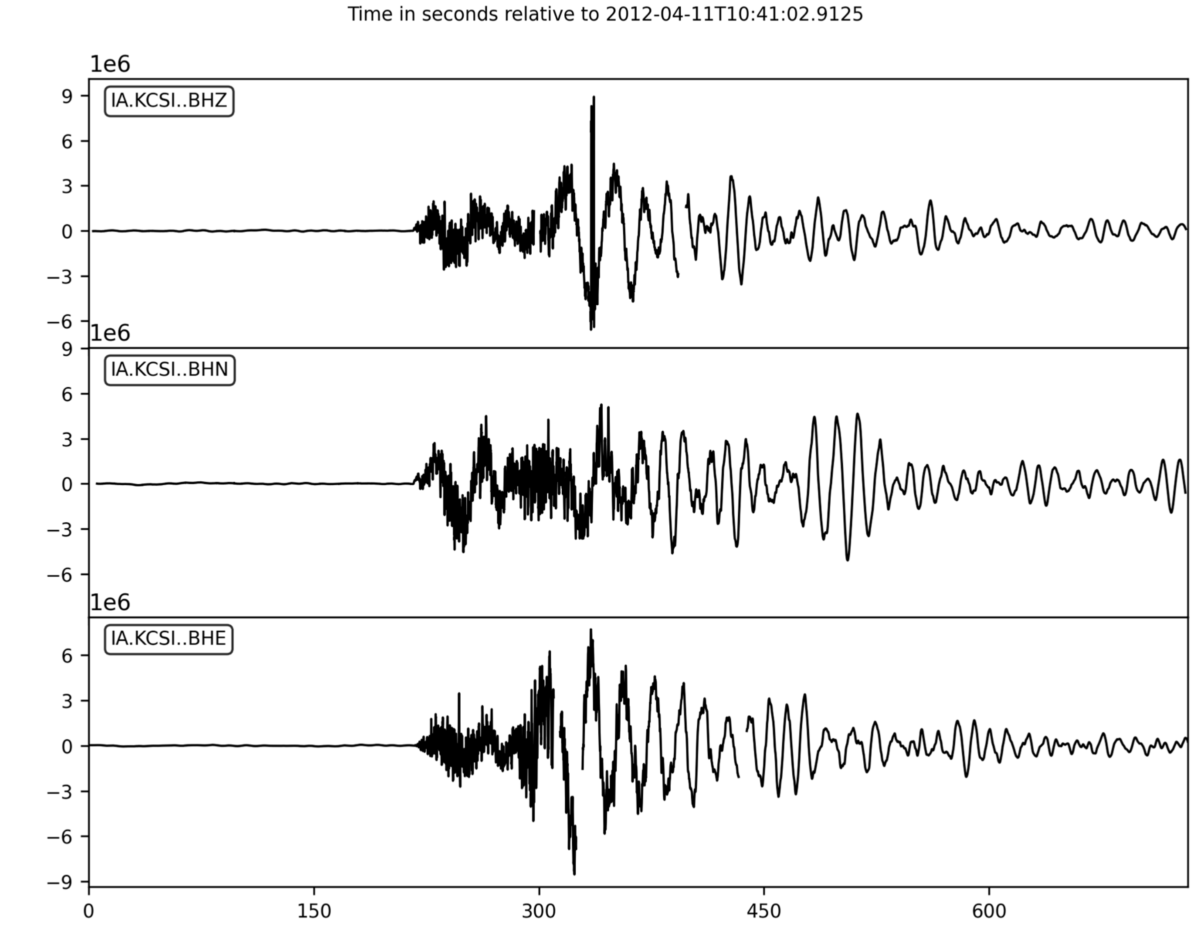
Time in seconds relative to 2012-04-11T10:41:02.9125
1e6
IA.KCSI..BHZ
IA.KCSI..BHN
IA.KCSI..BHE
9
6
3
0
−3
−6
−9
0
150
300
450
600

(c)

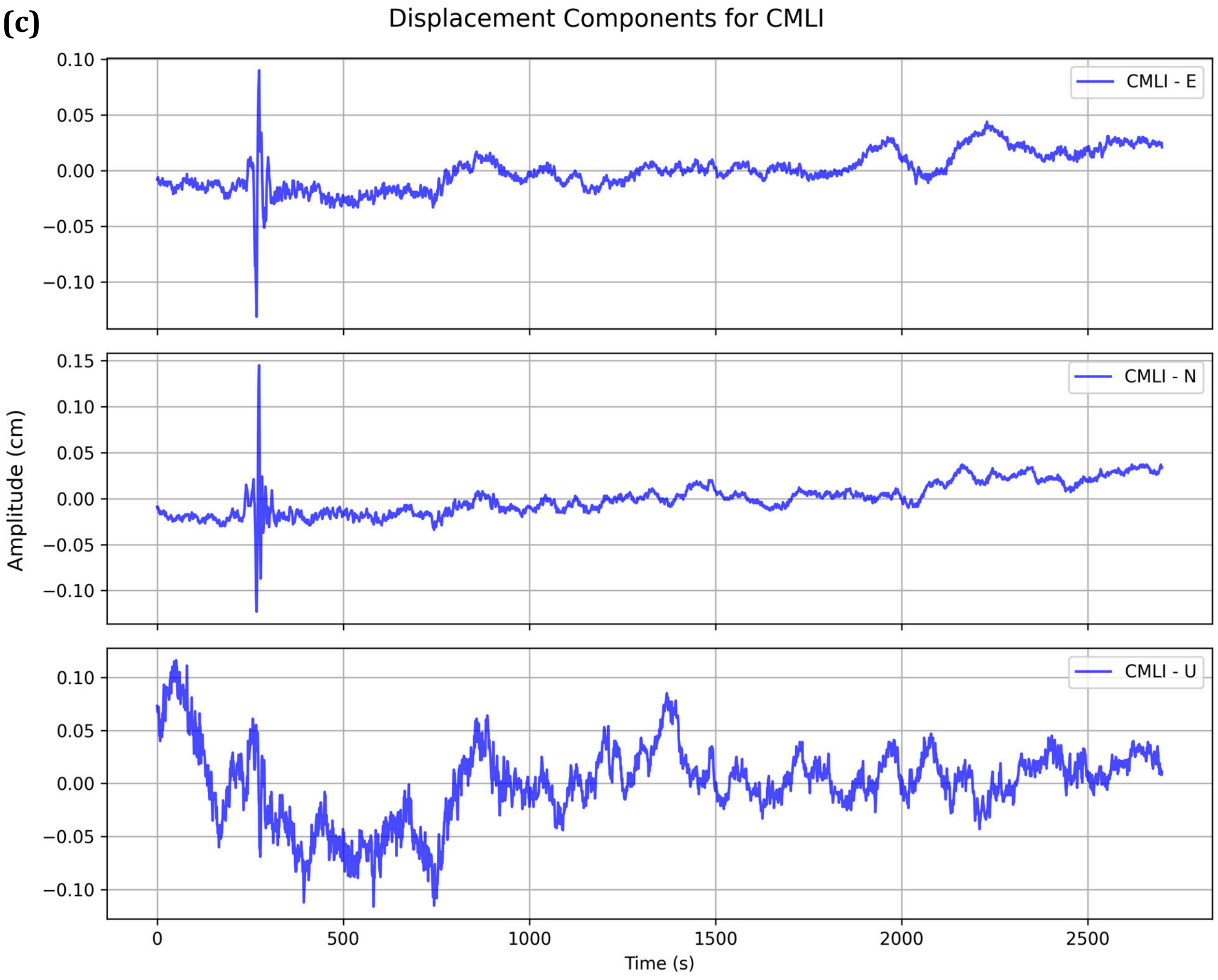
Displacement Components for CMLI
CMLI - E
CMLI - N
CMLI - U
Amplitude (cm)
Time (s)

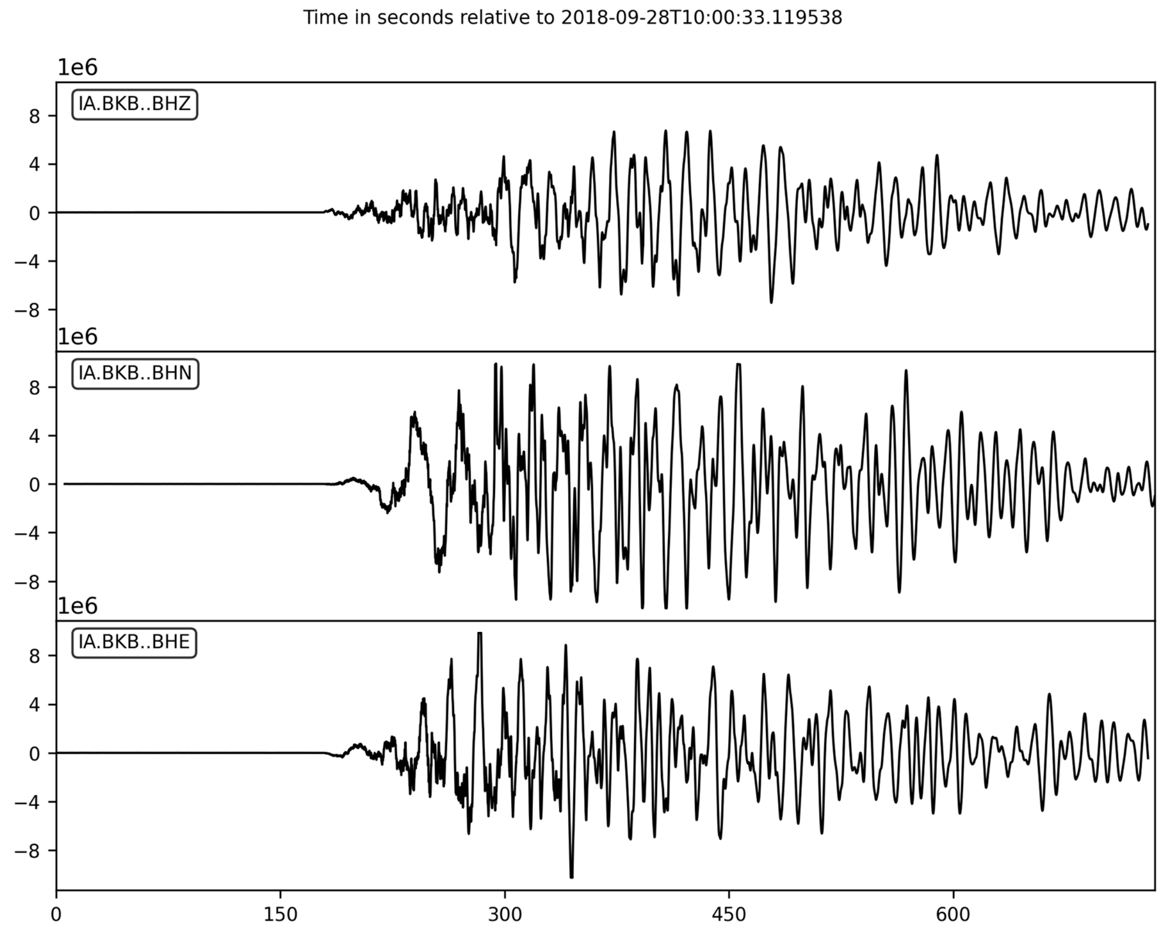
Time in seconds relative to 2018-09-28T10:00:33.119538
IA.BKB..BHZ
IA.BKB..BHN
IA.BKB..BHE
1e6

Figure S5: Comparison of Wavelet-Based and STA/LTA Methods for P-Wave Detection at PSKI (2009 Padang Earthquake, Mw 7.6). (a) North component GNSS waveform at PSKI (69.62 km), with wavelet-based P-wave detection at 10:16:56 UTC (+30.0 s offset, Table 1). (b) Wavelet power (blue) and dynamic threshold (yellow), showing the P-wave onset at 10:16:56 UTC where the wavelet power exceeds the threshold. (c) STA/LTA output (short-term window 5 s, long-term window 30 s, threshold 3.0), showing detection at 10:07:55 UTC (-522.6 s offset), demonstrating the wavelet method's superior robustness in low-SNR GNSS data.

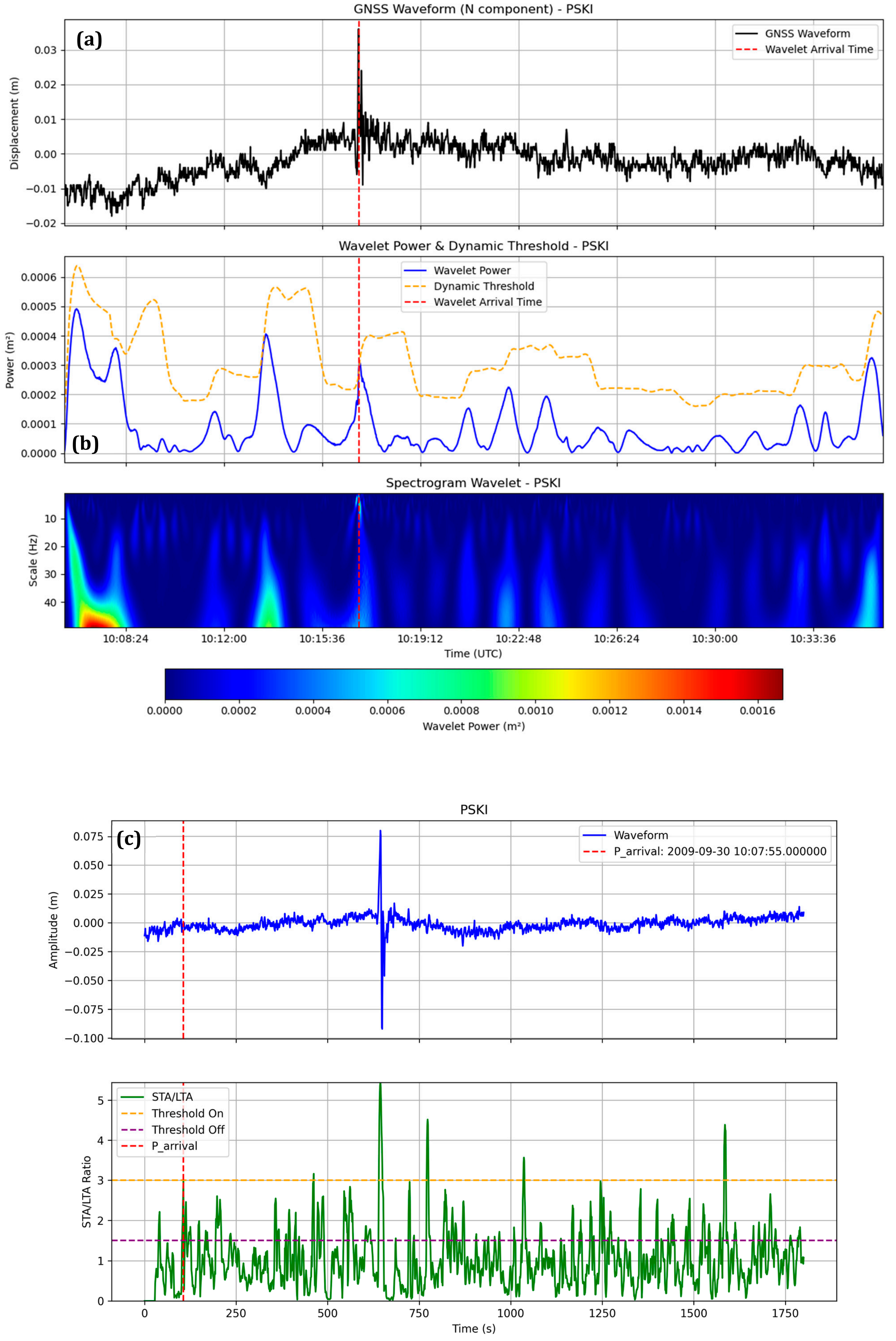

Figure S6: Comparison of Wavelet-Based and STA/LTA Methods for P-Wave Detection at BITI (2012 Simeulue Earthquake, Mw 8.6). (a) North component GNSS waveform at BITI (545.94 km), with wavelet-based P-wave detection at 08:39:30 UTC (−17.9 s offset, Table 1). (b) Wavelet power (blue) and dynamic threshold (yellow), showing the P-wave onset at 08:39:30 UTC where the wavelet power exceeds the threshold. (c) STA/LTA output (short-term window 5 s, long-term window 30 s, threshold 3.0), showing an invalid detection at 08:31:12 UTC (−515.9 s offset), highlighting the wavelet method's superior ability to detect P-waves in noisy GNSS data where STA/LTA provides an inaccurate result.

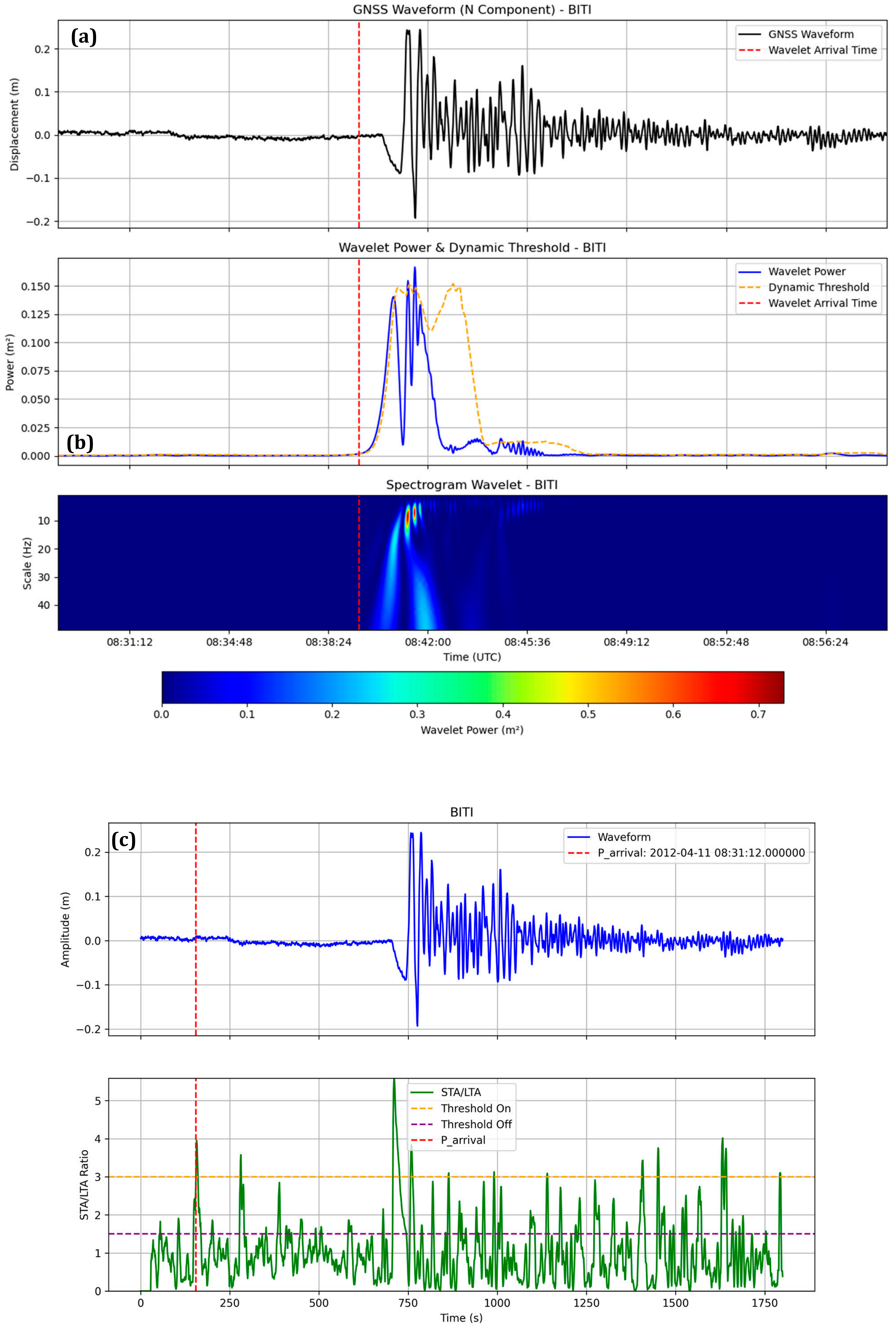

Figure S7: Comparison of Wavelet-Based and STA/LTA Methods for P-Wave Detection at CBAL (2018 Palu Earthquake, Mw 7.5). (a) North component GNSS waveform at CBAL (352.43 km), with wavelet-based P-wave detection at 10:04:39 UTC (+67.8 s offset, Table 1). (b) Wavelet power (blue) and dynamic threshold (yellow), showing the P-wave onset at 10:04:39 UTC where the wavelet power exceeds the threshold. (c) STA/LTA output (short-term window 1 s, long-term window 10 s, threshold 3.0), showing an early detection at 10:02:15 UTC (-76.2 s offset), highlighting the wavelet method's superior robustness in low-SNR GNSS data where STA/LTA provides a less accurate detection.

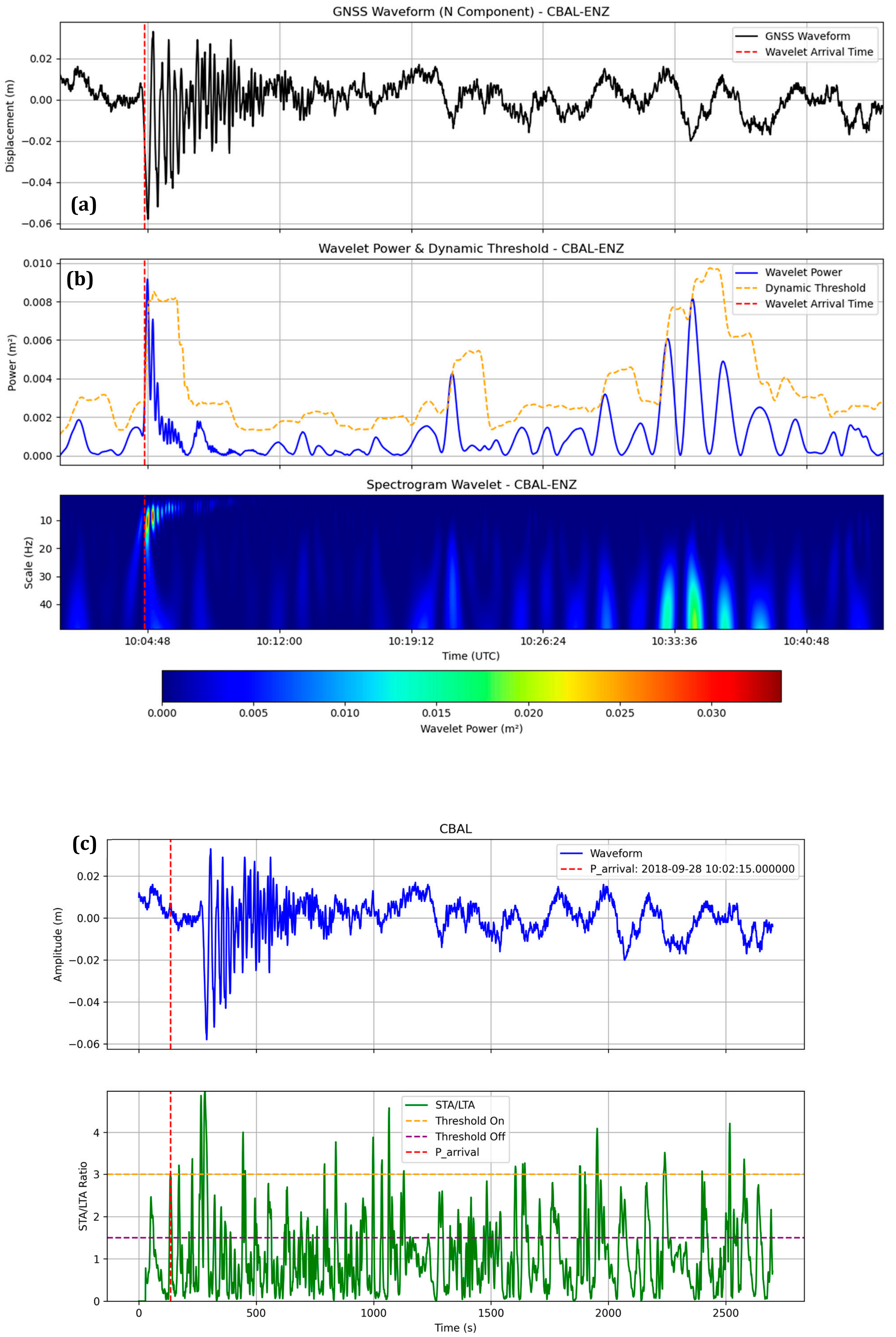

Texts

Text S1: Technical Details of P-wave Detection

This supplementary text provides the mathematical framework and technical details for the wavelet-based P-wave detection method applied to high-rate GNSS data, as described in Section 2.3 of the main manuscript. The method uses a Continuous Wavelet Transform (CWT) with a Mexican Hat wavelet to decompose GNSS displacement time series into the time-frequency domain, enabling robust detection of P-wave onsets in low signal-to-noise ratio (SNR) environments. The following equations outline the key steps, with parameters optimized for 1 Hz GNSS data.

Equation S1: Continuous Wavelet Transform (CWT)

$$W(a,b) = \frac{1}{\sqrt{a}} \int_{-\infty}^{\infty} x(t) \psi^* \left(\frac{t-b}{a} \right) dt$$

where ($W(a,b)$) is the wavelet coefficient, (a) is the scale parameter, (b) is the time shift, is the input GNSS displacement signal, and $\psi^*(t)$ is the complex conjugate of the Mexican Hat wavelet, defined as:

$$\psi(t) = \frac{2}{\sqrt{3}\pi^{1/4}} (1 - t^2) e^{-t^2/2}$$

Equation S2: Wavelet Power

$$P(a,t) = |W(a,b)|^2$$

Wavelet power represents the signal energy at each scale and time.

Equation S3: Mean Power Across Scales

$$P_{mean}(t) = \frac{1}{N_a} \sum_{a=1}^{N_a} P(a,t)$$

where $N_a = 50$ is the number of scales.

Equation S4: Rolling Standard Deviation

$$\sigma_{rool}(t) = \sqrt{\frac{1}{W} \sum_{i=t-W+1}^{t} (P_{mean}(i) - \mu_t)^2}$$

where W = 60 s is the window length, and μ_t is the mean power in the window.

Equation S5: Dynamic Threshold

$$T_{dyn}(t) = \mu_{pre} + k.\sigma_{roll}(t)$$

where μ_{pre} is the pre-event mean power, and k = 3.0 is the sensitivity factor, optimized to balance false positives and detection sensitivity (Section 2.3, Lines 161–165).

Equation S6: Arrival Time Estimation

$$t_{arrival} = t_{start} + \frac{i_{arrival}}{f_s}$$

where $f_s = 1\,Hz$ is the sampling frequency.

Equation S7: Index of Arrival Time

$$i_{arrival} = min\{i \mid P_{mean}(i) > T_{dyn}(i), i < i_{peak}\}$$

Identifies the earliest time when mean power exceeds the dynamic threshold.

Equation S8: Time Difference between GNSS and Seismic P-wave Arrivals

$$\Delta t = t_{arrival\ GNSS} - t_{arrival\ seismic}$$

Parameter Details: The scale range (1–50) corresponds to pseudo-frequencies from ~30 Hz to 0.6 Hz, with P-wave signals analyzed in the 0.02–0.5 Hz band (Page 11, Line 345). The sensitivity factor k = 3.0 was selected after testing values from 2.0 to 4.0, as lower values increased false positives and higher values missed weak signals (Page 4, Lines 142–174). The method was implemented using Python libraries (NumPy, PyWavelets, ObsPy) with custom configurations for GNSS data.
